# Lack of miR-378 attenuates muscular dystrophy in *mdx* mice

**DOI:** 10.1172/jci.insight.135576

**Published:** 2020-06-04

**Authors:** Paulina Podkalicka, Olga Mucha, Iwona Bronisz-Budzyńska, Magdalena Kozakowska, Katarzyna Pietraszek-Gremplewicz, Anna Cetnarowska, Urszula Głowniak-Kwitek, Karolina Bukowska-Strakova, Maciej Cieśla, Maria Kulecka, Jerzy Ostrowski, Michał Mikuła, Anna Potulska-Chromik, Anna Kostera-Pruszczyk, Alicja Józkowicz, Agnieszka Łoboda, Józef Dulak

**Affiliations:** 1 Department of Medical Biotechnology, Faculty of Biochemistry, Biophysics and Biotechnology, and; 2 Department of Clinical Immunology and Transplantology, Institute of Pediatrics, Medical College, Jagiellonian University, Krakow, Poland.; 3 Department of Gastroenterology, Hepatology and Clinical Oncology, Centre of Postgraduate Medical Education, Warsaw, Poland.; 4 Department of Genetics, Maria Sklodowska-Curie Memorial Cancer Center and Institute of Oncology, Warsaw, Poland,; 5 Department of Neurology, Medical University of Warsaw, Warsaw, Poland.

**Keywords:** Muscle Biology, Genetic diseases, Muscle, Noncoding RNAs

## Abstract

The severity of Duchenne muscular dystrophy (DMD), an incurable disease caused by the lack of dystrophin, might be modulated by different factors, including miRNAs. Among them, miR-378 is considered of high importance for muscle biology, but intriguingly, its role in DMD and its murine model (*mdx* mice) has not been thoroughly addressed so far. Here, we demonstrate that dystrophic mice additionally globally lacking miR-378 (double-KO [dKO] animals) exhibited better physical performance and improved absolute muscle force compared with *mdx* mice. Accordingly, markers of muscle damage in serum were significantly decreased in dKO mice, accompanied by diminished inflammation, fibrosis, and reduced abundance of regenerating fibers within muscles. The lack of miR-378 also normalized the aggravated fusion of dystrophin-deficient muscle satellite cells (mSCs). RNA sequencing of gastrocnemius muscle transcriptome revealed fibroblast growth factor 1 (*Fgf1*) as one of the most significantly downregulated genes in mice devoid of miR-378, indicating FGF1 as one of the mediators of changes driven by the lack of miR-378. In conclusion, we suggest that targeting miR-378 has the potential to ameliorate DMD pathology.

## Abstract 2

miR-378 deficiency improves the dystrophic phenotype in mdx mice, suggesting that modulation of miR-378 level may be a potential approach to attenuate DMD severity.

## Graphical abstract

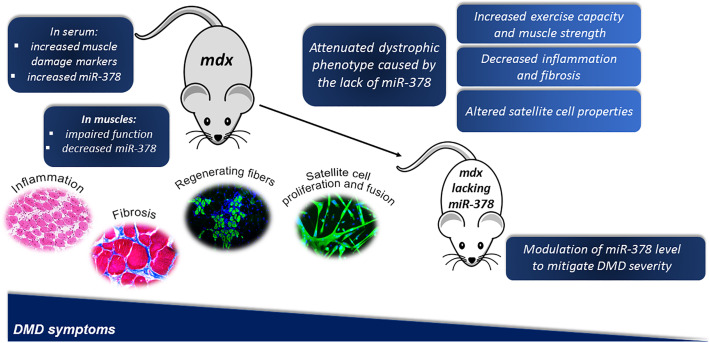

## Introduction

Duchenne muscular dystrophy (DMD, OMIM #310200), an X-linked genetic disorder that affects approximately 1 in 5000 boys, represents one of the most severe, devastating, and incurable forms of dystrophinopathies (1, 2). The lack of dystrophin — the crucial component of the muscle cytoskeleton, due to the over 7000 patient–specific mutations — leads to the mechanical instability and destruction of the myofibers upon contraction (3, 4). Concomitantly, myofibers degeneration triggers a strong and persistent inflammatory reaction, one of the major hallmarks of DMD pathology (5). In response to muscle deterioration, the muscle satellite cells (mSCs) (i.e., bona fide muscle stem cells) get activated, proliferate, and differentiate into myotubes to give rise to new muscle fibers (6). Nonetheless, dystrophin deficiency causes disturbed cell division and differentiation of mSCs. Moreover, repeated cycles of muscle damage and regeneration over the years result in the exhaustion of mSCs (7, 8). Consequently, the replacement of muscles by fibrous and fatty connective tissues appear (9). Eventually, patients suffering from DMD lose the ability to walk around the age of 12 and ultimately die in the second to third decade of life, predominantly due to cardiac or respiratory failure (10, 11).

Even though the primary cause of DMD is known already for more than 30 years, it remains an incurable disease. Hence, there is a constant need for creating novel therapeutic approaches aimed to at least alleviate the disease severity (12). Recently, our group reported the important role of heme oxygenase-1 (*Hmox1*, HO-1), a master cytoprotective and antiinflammatory enzyme, in the murine model of DMD, *mdx* mice. Whereas we demonstrated elevated levels of HO-1 in the muscles of dystrophic animals, its genetic or chemical inhibition significantly exacerbated dystrophic phenotype and influenced mSC properties, pointing toward the protective role of HO-1 in DMD progression (13). Moreover, we previously revealed HO-1 as a modulator of miRNA processing in vitro in the C2C12 myoblasts cell line (14). This prompted us to further expand the contribution of specific miRNAs in the pathology of DMD, taking into account its complexity and the ability of miRNAs to influence multiple biological processes at once. In line with that, dysregulation of miRNA expression has already been attributed to various skeletal muscle disorders, including muscular dystrophies, in which several miRNAs — such as miR-206, miR-499, and miR-208b — have been suggested as circulating biomarkers of DMD, thereby outperforming invasive muscle biopsies (15, 16).

Among miRNAs, miR-378a (miR-378) might be of particular importance in muscular dystrophy, and it was already reported to be highly abundant in muscles in comparison with other tissues (17, 18). Two mature strands, miR–378-3p and miR–378-5p, originate from the first intron of the peroxisome proliferator–activated receptor-γ-coactivator 1 β gene (*Ppargc1b*), encoding PGC-1β, a key regulator of energy metabolism (19). In skeletal muscles, miR-378 was described as a mediator of differentiation and proliferation of myoblasts through inhibition of musculin, the myogenic repressor (20).

Relevant in the context of DMD, miR-378 was reported to be downregulated in the muscles of dystrophic animals and DMD patients (18, 21). Simultaneously, it was demonstrated as one of the most upregulated miRNAs in the serum of different mouse models of the disease, as well as in DMD patients, emphasizing its potential utility as a circulating biomarker (22, 23). Even more, Zeng et al. demonstrated that overexpression of miR-378 attenuated muscle regeneration by delaying mSC activation and differentiation upon cardiotoxin-induced muscle injury (24). Therefore, we sought to explore the role of miR-378 loss in a murine model of DMD, *mdx* mice, with the special emphasis on pathobiology and severity of the hallmark symptoms of the disease.

## Results

### Dystrophic mice devoid of miR-378 demonstrate improved exercise capacity and absolute muscle force, accompanied by lean phenotype.

To thoroughly investigate the impact of miR-378 on disease severity in a commonly used mouse model of DMD, *mdx* mice, we generated double KO (dKO) animals devoid of both dystrophin and miR-378 that were directly compared with their *mdx* counterparts. Additionally, *mdx* mice were analyzed versus WT mice, and the comparison of miR-378–KO (miR-378^–/–^) veresus WT mice was studied, as well.

**Figure 1:**
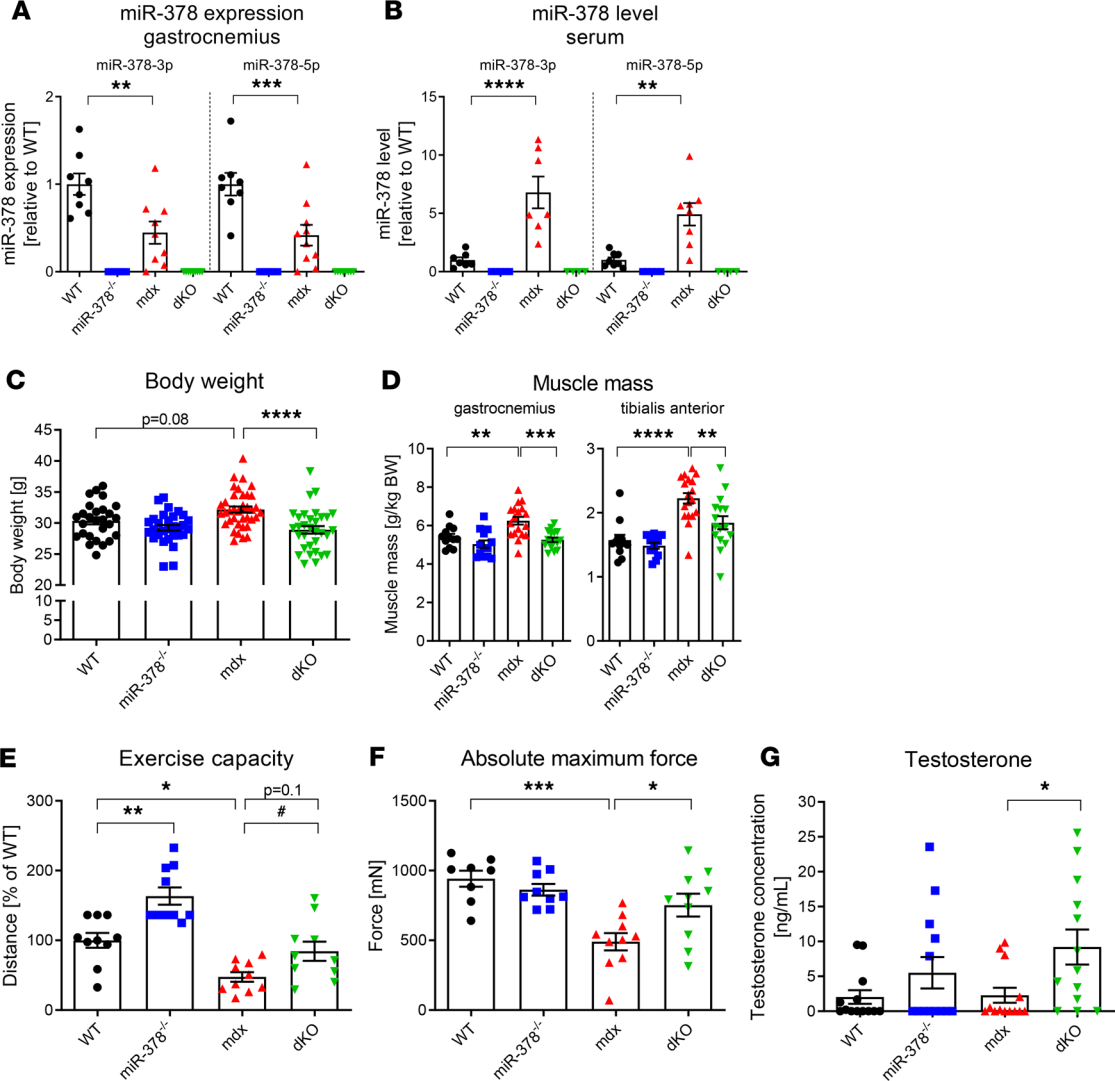
The lack of miR-378 improves running capacity and muscle strength in 3-month-old *mdx* mice. (**A** and **B**) The expression of miR–378-3p and miR–378-5p showing a decreased level of miR-378 in the gastrocnemius muscle (**A**) and increased level of miR-378 in the serum of dystrophic mice (**B**) with its undetectable expression in miR-378^–/–^ and dKO animals*;* qPCR; *n* = 4–10/group. (**C**) Decreased body weight of *mdx* mice lacking miR-378; *n* = 27–37/group. (**D**) Gastrocnemius and tibialis anterior muscle mass calculated per kg BW showing reduced muscle mass in dKO mice; *n* = 12–18/group. (**E**) Muscle performance suggesting the improved running capacity of dKO animals; downhill running treadmill test presented as the percentage of the running distance to exhaustion compared with WT animals; *n* = 10–11/group. (**F**) An increased absolute maximum force of tibialis anterior muscle of dKO animals; in situ muscle contractile measurements using the Aurora system; *n* = 8–10/group. (**G**) Elevated testosterone level in the serum of dKO animals; ELISA; *n* = 13/group. Data are presented as mean ± SEM. *P < 0.05; **P < 0.01; ***P < 0.001; ****P < 0.0001 by 1-way ANOVA with Tukey’s post hoc test; ^#^*P* < 0.05 with Student’s *t* test.

First, we checked the expression of miR-378 in gastrocnemius muscle of 3-month-old mice, and we observed lower levels of miR–378-3p and miR–378-5p in *mdx* animals (Figure 1A). Conversely, an elevation of both miR–378-3p and miR–378-5p in the serum of dystrophic animals was noticed (Figure 1B). In miR-378^–/–^ and dKO animals, miR-378 was undetectable, as expected (Figure 1, A and B).

The body weight of dKO animals was notably decreased (Figure 1C); additionally, the lack of miR-378 abolished muscle mass increase observed in gastrocnemius and tibialis anterior muscles of dystrophic animals (Figure 1D), suggesting increased fitness of mice lacking miR-378. Hence, it prompted us to assess muscle functionality by applying a downhill treadmill test and measuring muscle contractile properties in situ. As presumed, *mdx* mice could cover a shorter distance than the WT counterparts (Figure 1E). Surprisingly, miR-378^–/–^ mice exhibited higher running capacity than WT animals, while dKO animals ran similar distances to WT animals (Figure 1E). Importantly, dKO mice outperformed *mdx* animals as the improved absolute maximum force of the tibialis anterior muscle was visible (Figure 1F). Under scrutiny, the hyperactive behavior of mice devoid of miR-378 was evident, together with increased testosterone levels in the serum of dKO mice (Figure 1G).

### Mdx mice lacking miR-378 exhibit a lower level of muscle damage markers, together with attenuated inflammation.

**Figure 2:**
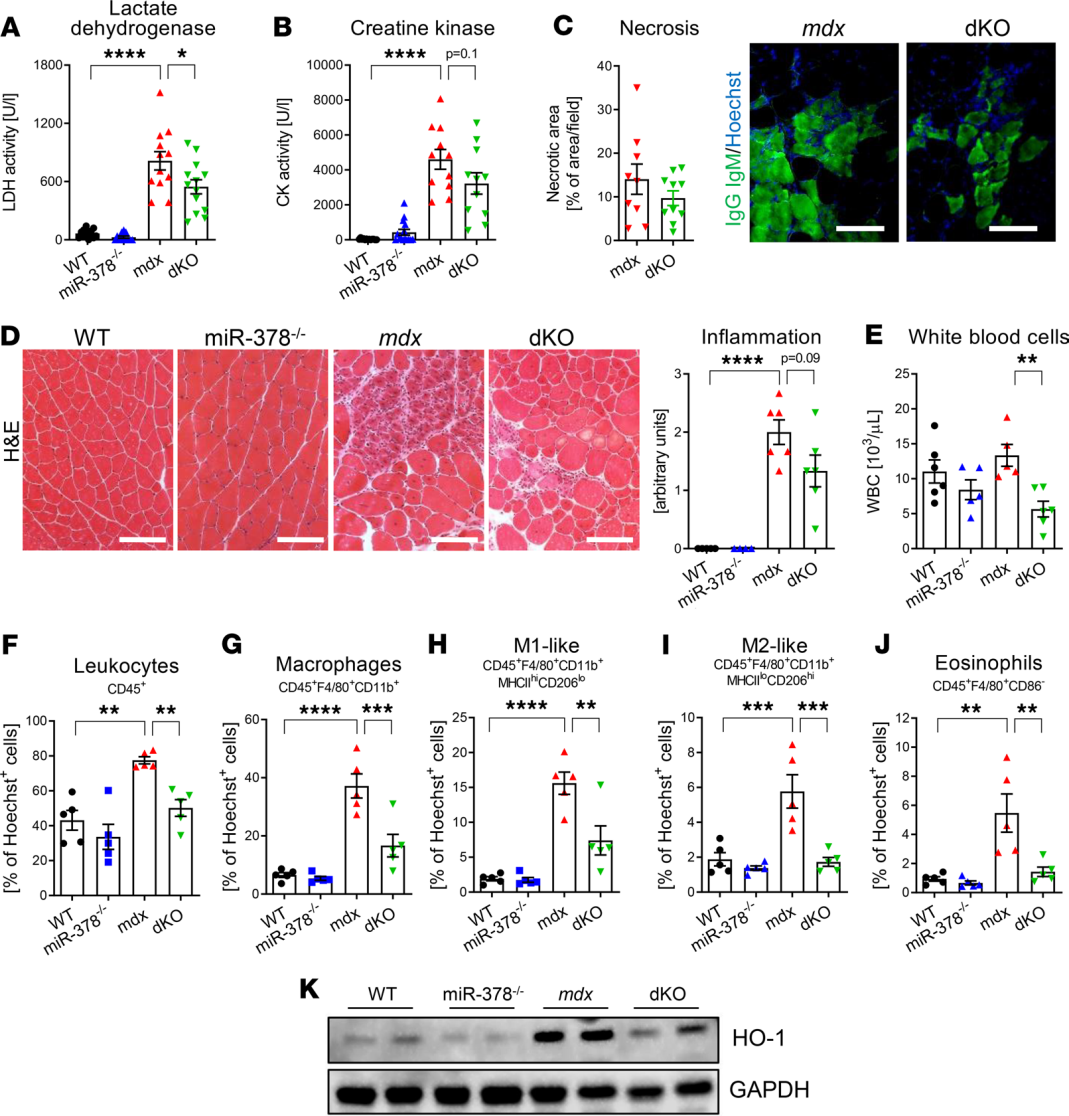
Muscle damage markers in serum and inflammation in gastrocnemius muscle are decreased in 3-month-old dystrophic mice lacking miR-378. (**A**) Lower serum activity of LDH in *mdx* animals lacking miR-378; activity assay; *n* = 10–14/group. (**B**) Increased serum CK activity in *mdx* mice with a tendency to be decreased by miR-378-KO, activity assay; *n* = 11–13/group. (**C**) Necrosis assessment by immunofluorescent staining of IgM and IgG (green) binding and its calculation indicating no differences between groups; *n* = 9–10/group. Scale bar: 100 μm. (**D**) Representative pictures of H&E staining of gastrocnemius muscle with semiquantitative analysis of inflammation extent showing a tendency in decreased inflammatory cell infiltration in dKO mice; microscopic assessment using Nikon Eclipse microscope. Scale bar: 100 μm; *n* = 4–6/group. (**E**) Decreased number of WBC in the peripheral blood in dKO mice; blood cell count; *n* = 5–6/group. (**F–J**) The analysis of inflammatory cells in hind limb muscles with special emphasis on macrophage subpopulations; flow cytometry analysis calculated as the percentage of Hoechst^+^ cells; *n* = 5/group. The percentage of CD45^+^ cells (**F**), macrophages (CD45^+^F4/80^+^CD11b^+^ cells) (**G**), M1-like macrophages (CD45^+^F4/80^+^CD11b^+^MHCII^hi^CD206^lo^ cells) (**H**), M2-like macrophages (CD45^+^F4/80^+^CD11b^+^MHCII^lo^CD206^hi^ cells) (**I**), and eosinophils (CD45^+^F4/80^+^CD86^+^ cells) (**J**) showing significant decrease in dKO mice. (**K**) The decreased HO-1 protein level in dKO animals assessed by Western blot; GAPDH used as loading control. Representative result of 2 independent experiments; *n* = 4–5/group. Data are presented as mean ± SEM. *P < 0.05; **P < 0.01; ***P < 0.001; ****P < 0.0001; (**A–B** and **D–J**) 1-way ANOVA with Tukey’s post hoc test; (**C**) unpaired 2-tailed Student’s *t* test.

To further explore the impact of miR-378 loss on hallmark symptoms of DMD, we aimed to investigate the muscle damage markers and inflammation extent. It appeared that the lactate dehydrogenase (LDH) activity in *mdx* mice lacking miR-378 was diminished when compared with *mdx* mice (Figure 2A). Accordingly, creatine kinase (CK) levels in dKO mice versus *mdx* tended to be decreased (Figure 2B). However, the lack of miR-378 did not attenuate gastrocnemius muscle necrosis of dystrophic mice, as assessed by the immunofluorescent staining of the IgG/IgM, the membrane-impermeable serum markers (Figure 2C) (25).

H&E staining assessment revealed a massive accumulation of inflammatory cells in the gastrocnemius muscle of *mdx* mice (Figure 2D). Interestingly, in dKO animals at the same age, slightly lower inflammatory infiltration was detected (Figure 2D), followed by a decreased number of WBC in the peripheral blood of dKO mice (Figure 2E). Interestingly, no severe abnormalities in the level of WBC were found in the peripheral blood of dystrophic animals (Figure 2E), consistent with the data obtained from DMD patients (Supplemental Table 1; supplemental material available online with this article; https://doi.org/10.1172/jci.insight.135576DS1).

To better dissect the contribution of specific subsets of immune cells toward observed effects, flow cytometry analysis on the hind limb muscles was performed (Figure 1, F–J, and Supplemental Figure 1). That included both the bulk of CD45^+^ blood cells, as well as a more refined analysis of eosinophil and macrophage subtypes. The latter are essential mediators of myogenic regeneration (26), and miR–378-3p was recently described to be involved in their activation (27). The results unequivocally show a significant reduction in the percentage of immune cells (Figure 2F) in muscles of dKO mice, which further strengthened our histological assessment. Strikingly, we revealed that percentage of CD45^+^F4/80^+^CD11b^+^ macrophages (Figure 2G) — as well as both proinflammatory M1-like (CD45^+^F4/80^+^CD11b^+^MHCII^hi^CD206^lo^) and antiinflammatory M2-like (CD45^+^F4/80^+^CD11b^+^MHCII^lo^CD206^hi^) subpopulations (Figure 2, H and I, respectively) — were diminished in dKO mice. Apart from macrophages, eosinophils also have been reported to significantly contribute to the pathology of muscular dystrophy. Their elevated level was shown to promote the progression of DMD by, among others, increasing fibrosis in skeletal and heart muscles of *mdx* mice (28, 29). Indeed, the number of eosinophils (CD45^+^F4/80^+^CD86^–^ immunophenotype) was markedly increased in the muscles of *mdx* mice (Figure 2J). Of note, the lack of miR-378 in dystrophic animals reduced eosinophils’ abundance (Figure 2J), further indicating that those mice exhibit overall decreased inflammation. Finally, the level of damage-induced antiinflammatory HO-1 was potently elevated in muscles of *mdx* animals, confirming our previous results (13), with a concomitant decrease in dystrophic mice lacking miR-378 (Figure 2K).

### Muscle fibrosis is decreased in dystrophic mice lacking miR-378.

**Figure 3:**
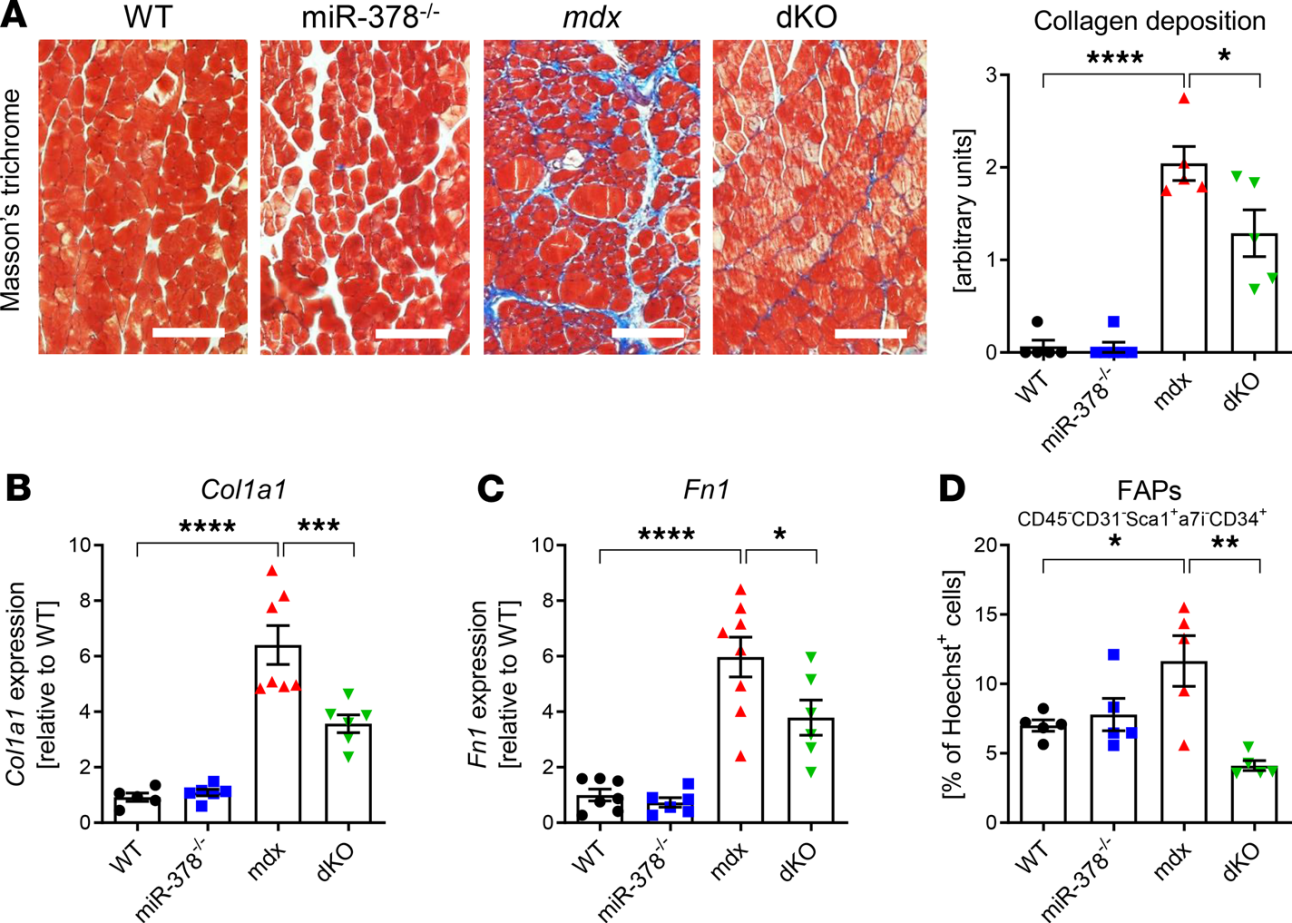
Fibrosis extent is diminished in gastrocnemius muscle of 3-month-old dystrophic mice lacking miR-378. (**A**) Representative photos of Masson’s trichrome staining with semiquantitative analysis of collagen deposition showing the decreased extent of fibrosis in dKO mice; microscopic assessment using Nikon Eclipse microscope. Scale bar: 100 μm; *n* = 5–6/group. (**B** and **C**) Decreased expression of fibrotic markers in dKO mice: *Col1a1* (**B**) and *Fn1* (**C**), qPCR; *n* = 5–8/group. (**D**) The diminished abundance of FAPs identified as CD45^–^CD31^–^Sca1^+^α7i^–^CD34^+^ cells in hind limb muscles of dKO mice; flow cytometry analysis calculated as the percentage of Hoechst^+^ cells; *n* = 5/group. Data are presented as mean ± SEM. *P < 0.05; **P < 0.01; ***P < 0.001; ****P < 0.0001; 1-way ANOVA with Tukey’s post hoc test.

During DMD progression, extensive fibrosis and adipogenic degeneration occur in the affected muscle tissues (30). Accordingly, we demonstrate that, in dystrophic gastrocnemius muscles, the fibrosis extent was significantly increased; however, the lack of miR-378 in dystrophic mice led to decreased collagen deposition in gastrocnemius muscle (Figure 3A). Simultaneously, reduced expression of fibrotic markers, collagen type I α 1 (*Col1a1*) and fibronectin 1 (*Fn1*) was noticed in dKO animals (Figure 3, B and C). Furthermore, since fibro/adipogenic progenitors (FAPs) are also involved in the progression of DMD (31), the flow cytometry analysis of this population, defined as CD45^–^CD31^–^Sca1^+^α7i^–^CD34^+^, was performed (Supplemental Figure 2). Interestingly, in hind limb muscles of dKO animals, a prominent decrease in the percentage of FAPs was observed (Figure 3D).

### miR-378 loss affects the properties of dystrophic mSCs.

**Figure 4:**
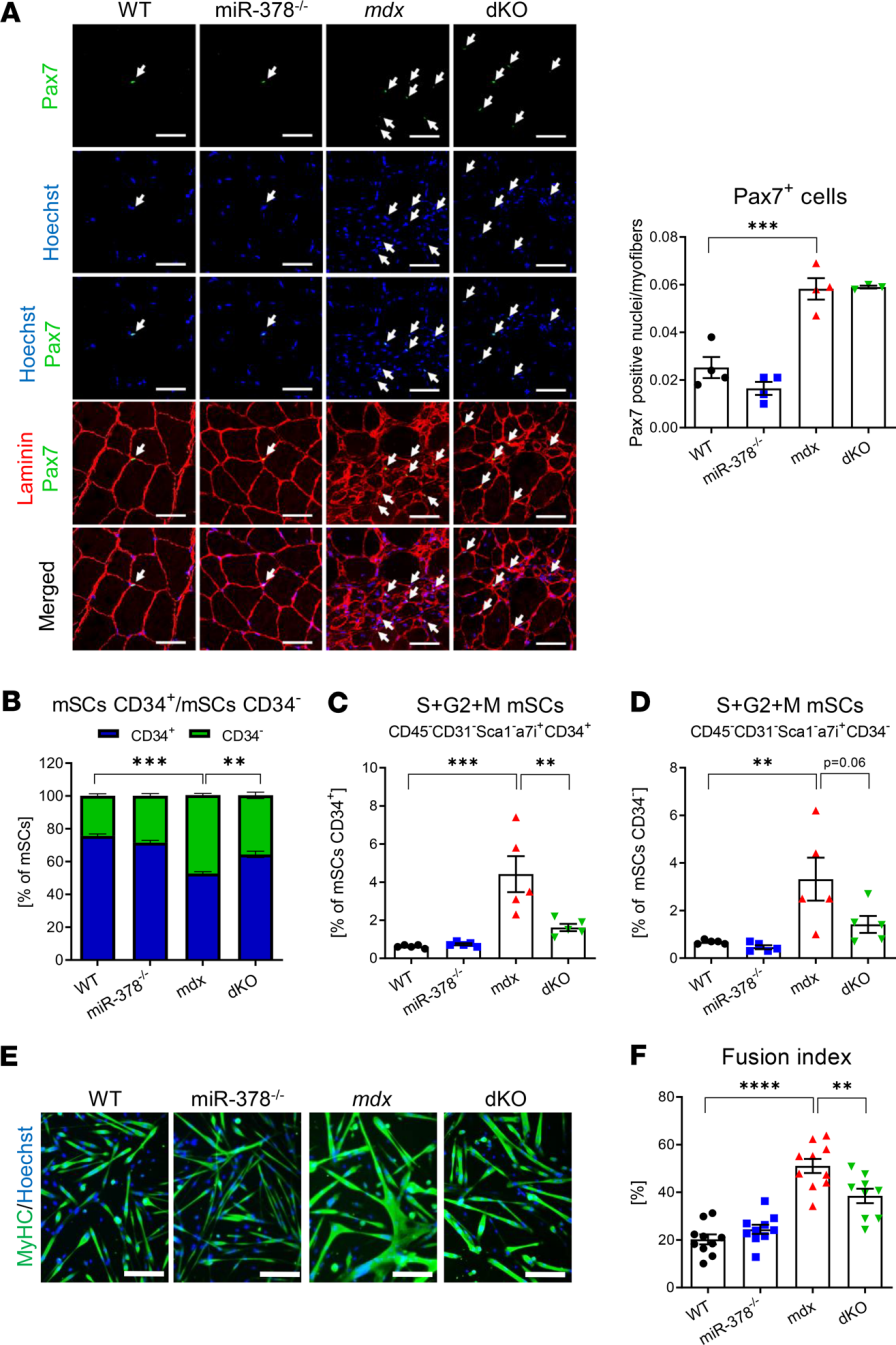
The KO of miR-378 affects the phenotype and properties of dystrophic muscle satellite cells (mSCs). (**A**) The abundance of mSCs within the gastrocnemius muscle of 3-month-old *mdx* mice without the apparent influence of miR-378, as quantified based on Pax7^+^ nuclei per myofibers. Immunofluorescent staining with representative pictures; confocal microscope LSM-510, Carl Zeiss. Scale bar: 50 μm; *n* = 3–4/group. Arrows indicate Pax7^+^ cells (green) colocalizing with nuclei stained with Hoechst (blue). (**B–D**) The analysis of mSCs in hind limb muscles of 10-week-old mice; flow cytometry analysis; *n* = 5/group. (**B**) quiescent (CD34^+^) and activated (CD34^–^) cells contribution within mSCs (CD45^–^CD31^–^Sca1^–^α7i^+^) population showing a decrease in CD34^–^ cells in dKO mice. The percentage of CD34^+^ (**C**) and CD34^–^ (**D**) mSCs in S + G2 + M phases of the cell cycle, revealing a decreased percentage of mSCs in the proliferative state of the cell cycle in dKO mice. (**E**) Representative pictures of MyHC (green) and Hoechst (blue) immunofluorescent staining of mSCs isolated from hind limb muscles of 10-week-old mice differentiating for 3 days ex vivo. Nikon Eclipse microscope. Scale bar: 100 μm; *n* = 9–10/group. (**F**) Fusion index determined by the percentage of MyHC^+^ fibers containing 3 or more nuclei among the total number of nuclei showing a significant decrease in the fusion index in dKO mice; *n* = 9–10/group. Data are presented as mean ± SEM. **P < 0.01; ***P < 0.001; ****P < 0.0001; (**A**, **C**, **D**, and **F**) 1-way ANOVA with Tukey’s post hoc test; (**B**) 2-way ANOVA with Tukey’s post hoc test.

As inflammation, fibrosis, and FAPs disturb muscle regeneration, which is primarily driven by mSCs (32), we suspected that the abundance and properties of mSCs in dystrophic mice devoid of miR-378 might be altered, as well. Whereas the absolute number of Pax7^+^ mSCs was significantly elevated in the gastrocnemius muscle of dystrophic animals, no apparent changes driven by the additional lack of miR-378 were visible (Figure 4A). Nonetheless, detailed, flow cytometry–based examination of the contribution of quiescent (CD45^–^CD31^–^Sca1^–^α7i^+^CD34^+^) and activated (CD45^–^CD31^–^Sca1^–^α7i^+^CD34^–^) mSCs toward whole mSCs pool (defined as CD45^–^CD31^–^Sca1-α7i^+^ cells) in the hind limb muscles demonstrated that the population of activated mSCs is markedly increased in *mdx* mice and subsequently decreased in dKO animals (Figure 4B and Supplemental Figure 2). Interestingly, we observed higher proliferation status of both quiescent (Figure 4C) and activated (Figure 4D) mSCs — the effect that was diminished in dKO animals, particularly in the case of quiescent mSCs (Figure 4C). Thus, obtained results indicate that the lack of miR-378 at least partially rescues the activated, dystrophic phenotype of mSCs.

Next, the bulk of activated and quiescent mSCs was isolated from hind limb muscles, and their ability to fuse and form myotubes ex vivo was assessed based on the immunofluorescence staining for myosin heavy chain (MyHC). After 3 days of culture in muscle differentiation–promoting conditions, we observed the formation of fused, elongated, and MyHC^+^ myotubes in WT mSCs, an effect greatly aggravated in the case of dystrophic mSCs, which formed much thicker, multinucleated tubes with multiple branching points (Figure 4E). Strikingly, differentiation of dKO mSCs resulted in the formation of structures more reminiscent of miR-378^–/–^ or WT, rather than *mdx* myotubes (Figure 4D). The quantitative analysis revealed an increased fusion index of *mdx* mSCs, which was subsequently diminished by the additional lack of miR-378 (Figure 4F).

### The influence of miR-378 on regeneration and angiogenesis markers in dystrophic mice.

**Figure 5:**
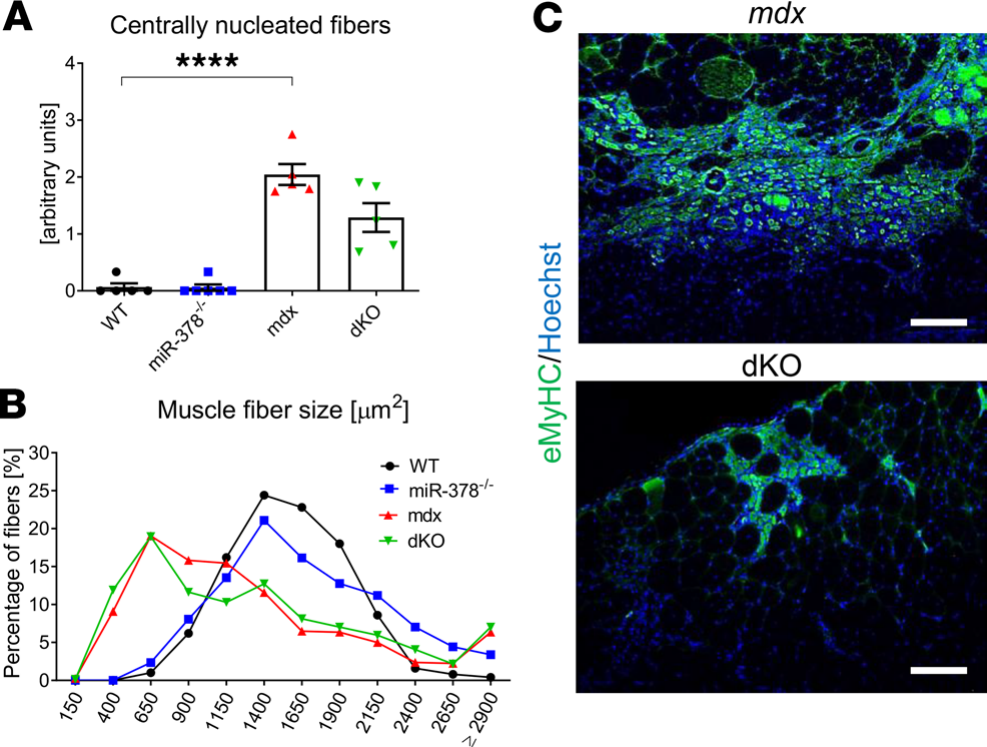
The influence of miR-378 loss on dystrophic muscle regeneration markers in gastrocnemius muscle of 3-month-old animals. (**A**) Semiquantitative analysis of centrally nucleated fibers performed based on H&E staining showing no differences between analyzed groups; *n* = 5–6/group. (**B**) Quantification of muscle fiber size based on laminin staining (not shown), indicating no effect of miR-378 deficiency; *n* = 4–5/group. (**C**) Immunofluorescent staining of eMyHC expression (green). Note the decreased abundance of eMyHC^+^ fibers in dKO mice; Hoechst (blue) was used to visualize nuclei; representative pictures with Nikon Eclipse microscope. Scale bar: 100 μm; *n* = 4–5/group. Data are presented as mean ± SEM. ****P < 0.0001; 1-way ANOVA with Tukey’s post hoc test.

Muscle regeneration is driven by activation of mSCs that proliferate, fuse, and differentiate to finally give rise to mature muscle fibers restoring homeostatic muscle after damage (33). Taking into account the decreased proliferation and differentiation capacity of dKO mSCs, we wondered if these alterations would be further reflected by the changes in muscle fibers regeneration capacity. Toward this end, we assessed the presence of centrally nucleated myofibers and the abundance of embryonic MyHC isoform–positive (eMyHC^+^) fibers (33–35). Semiquantitative analysis of centrally nucleated myofibers (Figure 5A), together with the muscle fiber size examination (Figure 5B) in gastrocnemius muscle, did not reveal any alterations driven by the lack of miR-378. However, we observed a marked decrease in the eMyHC^+^ fibers in dystrophic mice lacking miR-378 (Figure 5C), possibly indicative of the attenuated regeneration process in dystrophic mice devoid of miR-378. Similar results were obtained in soleus muscles (Supplemental Figure 3, A–C).

Muscle regeneration is accomplished by the formation of new blood vessels, and miR-378 was demonstrated to contribute to this process — for example, by regulating *Vegfa* expression (17, 36). Nonetheless, although VEGFA was markedly decreased in *mdx* versus WT animals, indicating possible alterations of angiogenesis, no differences in mice additionally lacking miR-378 were observed (Supplemental Figure 4A). Similarly, a diminished level of *Pecam1* (*Cd31*), a marker of blood vessels, was noticed in *mdx* versus WT animals without the apparent influence of miR-378 (Supplemental Figure 4B). Furthermore, no changes in the blood vessel abundance were revealed in dKO versus *mdx* animals (Supplemental Figure 4C), undermining the possible impact of miR-378 on vascularization under dystrophic conditions.

### RNA sequencing analysis of gastrocnemius muscle reveals Fgf1 as one of the possible mediators of miR-378.

**Figure 6:**
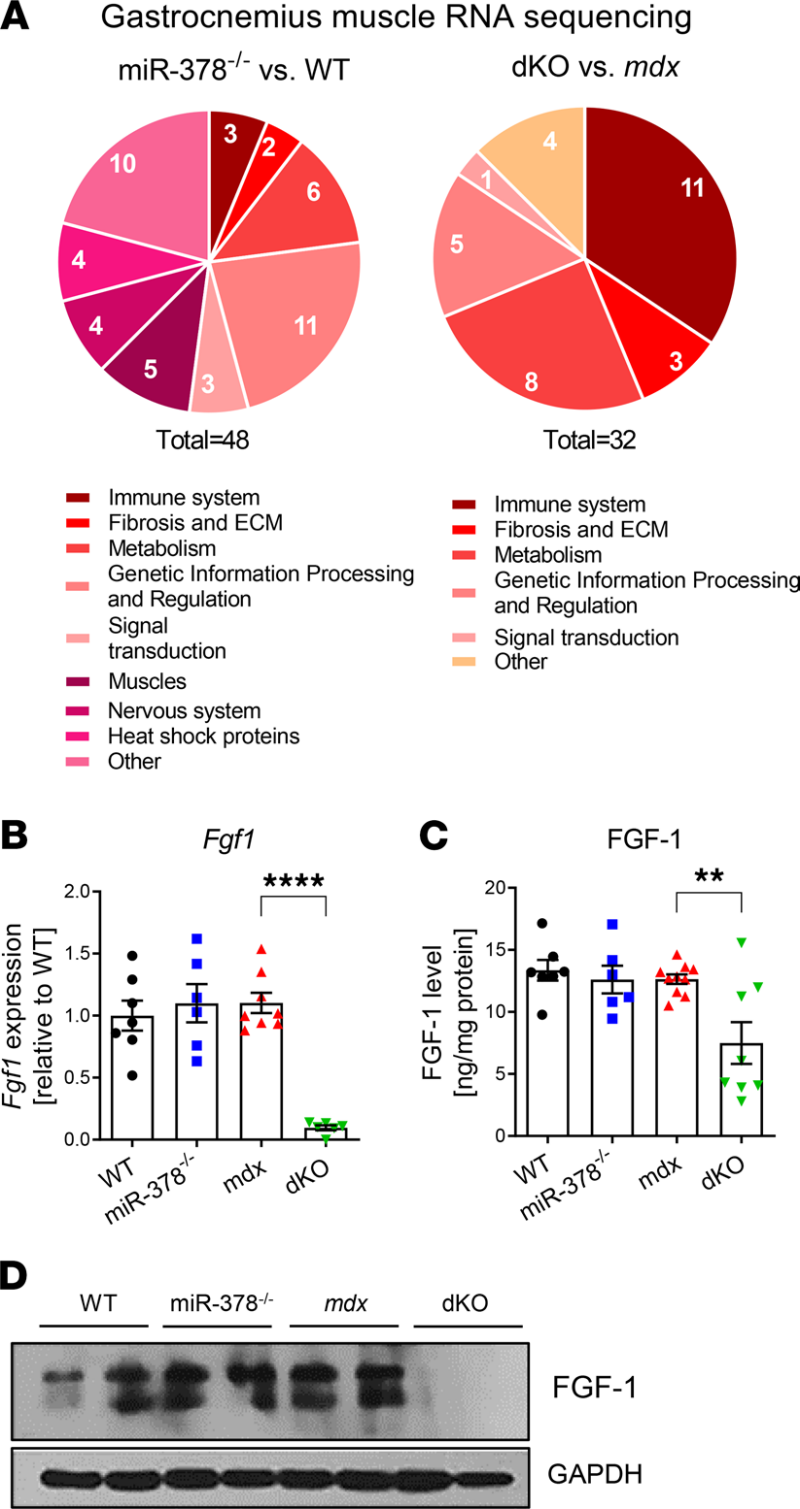
FGF1 is strongly decreased in dystrophic mice lacking miR-378. (**A**) Schematic representation of RNA sequencing results of gastrocnemius muscle from 3-month-old animals, showing genes differentially expressed in miR-378^–/–^ versus WT and dKO versus *mdx* animals with a specified contribution in different molecular processes. *n* = 4/group. (**B**) Significant decrease in *Fgf1* mRNA level in gastrocnemius muscle of dKO mice; qPCR; *n* = 6–8/group. (**C** and **D**) The markedly diminished FGF1 protein level in gastrocnemius muscle of dKO mice demonstrated by ELISA (**C**) and Western blot (**D**) analysis; *n* = 6–10/group. Data are presented as mean ± SEM. **P < 0.01; ****P < 0.0001; 1-way ANOVA with Tukey’s post hoc test.

To shed more light on possible mechanisms underlying an effect of miR-378 depletion, we performed comparative transcriptome analysis of gastrocnemius muscle from 3-month-old WT, *mdx*, miR-378^–/–^, and dKO animals. Surprisingly, the results showed only 48 differentially expressed genes in miR-378^–/–^ versus WT animals (Supplemental Table 2) and 32 in dKO versus *mdx* mice (Supplemental Table 3). However, these genes were enriched for transcripts related to the immune system, fibrosis, and extracellular matrix, as well as metabolism regulation (Figure 6A). The Kyoto Encyclopedia of Genes and Genomes (KEGG; https://www.genome.jp/kegg/) analysis revealed a decrease in the expression of genes related to antigen processing and presentation pathway in dKO mice (Supplemental Figure 5), providing a possible explanation for diminished inflammation observed in muscles lacking miR-378 (Figure 2).

Interestingly, we found *Fgf1* as one of the genes markedly reduced in muscles upon miR-378 depletion. We further confirmed a significant decrease in Fgf1 mRNA (Figure 6B) and protein (Figure 6, C and D) level, not only in gastrocnemius, but also in soleus (Supplemental Figure 3, D–F) muscle of dKO animals. A similar pattern of FGF1 expression was observed in cardiac muscle, but not in the liver, possibly suggesting FGF1 as a muscle-specific mediator of miR-378 action (P. Podkalicka [Jagiellonian University in Kraków, Poland], unpublished observations).

### The impact of miR-378 deficiency on muscle fiber type composition in dystrophic animals.

**Figure 7:**
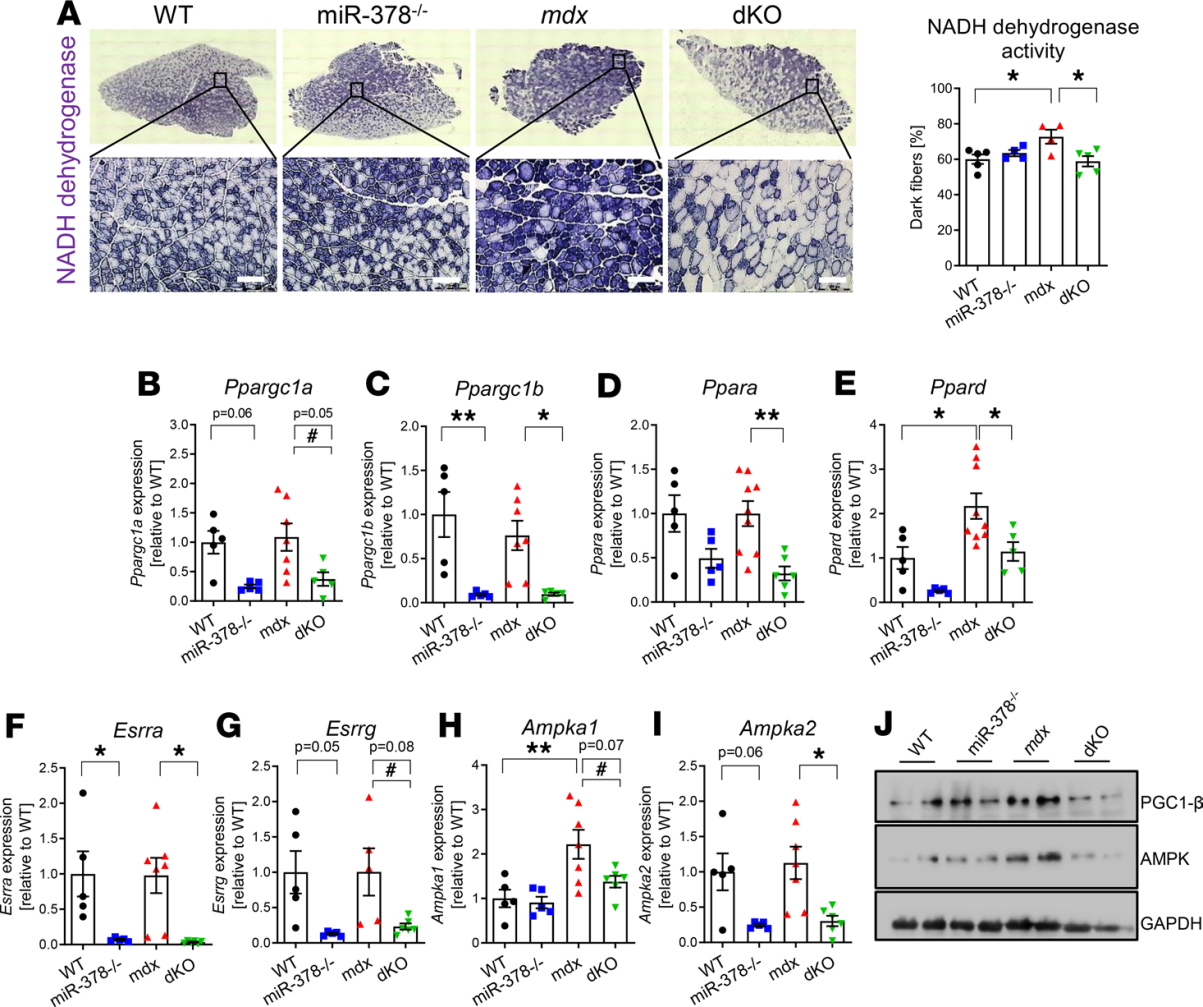
The impact of miR-378 deficiency on muscle fiber type composition and the expression of metabolic genes in the tibialis anterior muscle of 3-month-old mice. (**A**) Representative pictures of histochemical assessment of NADH dehydrogenase activity, indicating the increased percentage of slow (oxidative) fibers in *mdx* mice and the opposite effect in dKO animals; microscopic assessment using Nikon Eclipse microscope. Scale bar: 100 μm; *n* = 4–5/group. (**B–I**) The decreased expression of *Ppargc1a* (**B**), *Ppargc1b* (**C**), *Ppara* (**D**)*, Ppard* (**E**)*, Esrra* (**F**)*,* *Esrrg* (**G**)*,* *Ampka1* (**H**), and *Ampka2* (**I**) in dKO animals; qPCR; *n* = 5–7/group. (**J**) The diminished protein level of PGC1-β and AMPK in dKO mice assessed by Western blot; GAPDH was used as loading control. Representative result of 2 independent experiments; *n* = 4/group. Data are presented as mean ± SEM. *P < 0.05; **P < 0.01 by 1-way ANOVA with Tukey’s post hoc test; ^#^*P* < 0.05 with Student’s *t* test.

Skeletal muscles are heterogeneous tissue, composed of different types of muscle fibers. They can be distinguished based on their metabolic capacity, evidenced by stainings (e.g., NADH dehydrogenase, succinate dehydrogenase [SDH] activity, or various MyHC isoforms expression). Type I (MyHCI) marks slow-twitch fibers considered to be predominantly oxidative, and type II (MyHCII) distinguishes fast-twitch fibers that are more glycolytic (37). The contribution of oxidative versus glycolytic fibers might reflect the exercise capacity of the muscles, and in general, increased content of oxidative fibers promotes endurance phenotype (38). This prompted us to thoroughly investigate the possible alterations in fiber type composition driven by the lack of miR-378, especially because the influence of miR-378 on metabolism is well documented (19). Surprisingly, the analysis of tibialis anterior muscle, considered as a glycolytic muscle, revealed markedly increased oxidative fibers percentage in *mdx* animals — an effect that was reversed in dKO animals to the level observed in WT animals, as evidenced by NADH activity staining (Figure 7A). Interestingly, the metabolic makeup of different muscles seems to affect the response to the loss of miR-378. In a reportedly oxidative muscle, soleus, the percentage of type I fibers was potently elevated in *mdx* mice based on both immunofluorescent staining for MyHCI and SDH activity; however, no apparent changes in dystrophic mice devoid of miR-378 were noticeable (Supplemental Figure 3G). Furthermore, the analysis of genes associated with oxidative metabolism (39–41) in tibialis anterior muscle of dKO mice consistently revealed diminished mRNAs encoding *Ppargc1a*, *Ppargc1b*, *Ppara*, *Ppard*, *Esrra*, *Esrrg*, *Ampka1*, and *Ampka2* (Figure 7, B–I). Finally, changes in the protein levels of PGC1-β and AMPK in the tibialis anterior muscle of dKO mice (Figure 7J) further indicated the possible impact of miR-378 loss on metabolic-related pathways in dystrophic animals.

### The effect of miR-378 deficiency in the diaphragm muscle.

**Figure 8:**
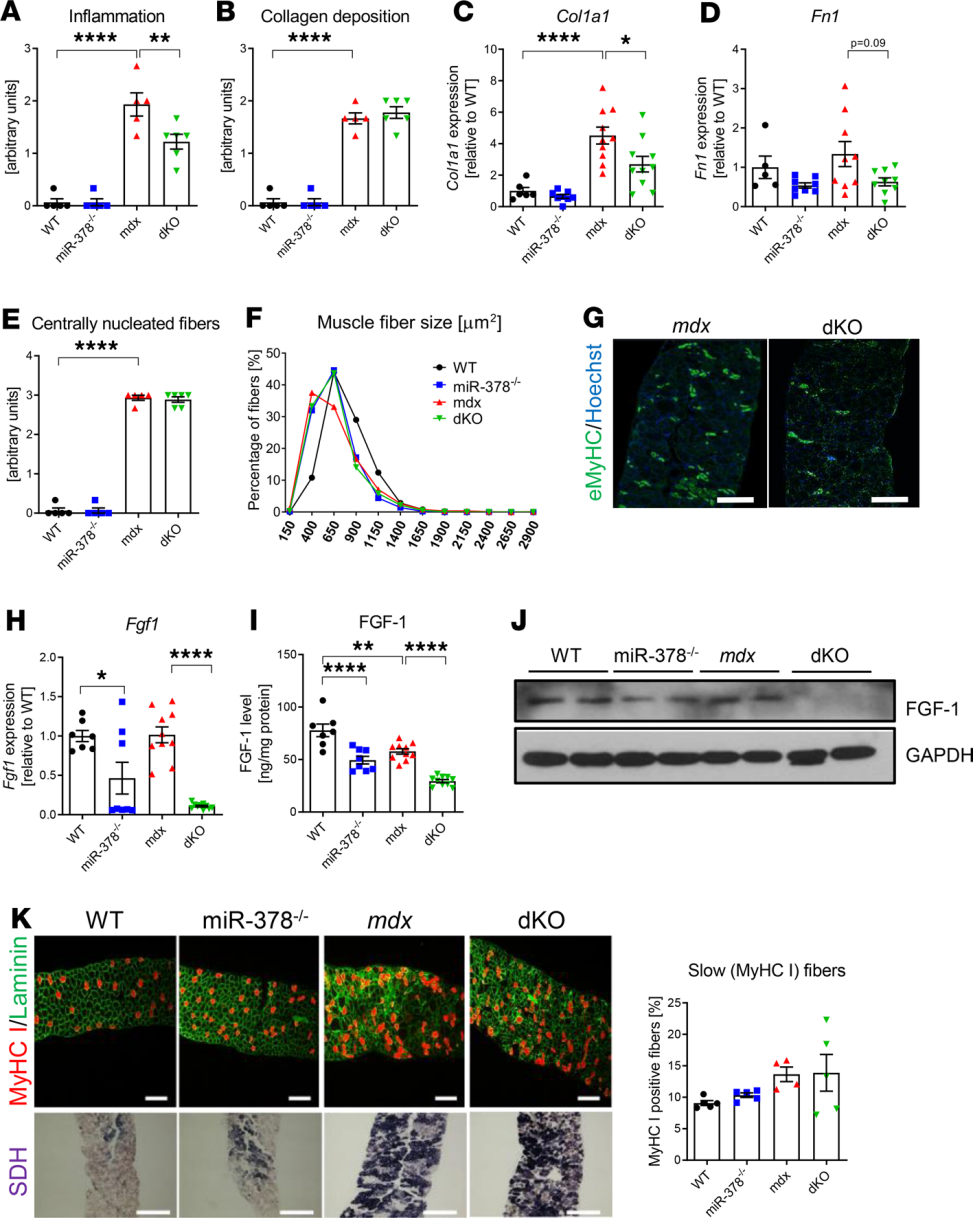
The effect of miR-378 deficiency is also evident in the diaphragm of 3-month-old animals. (**A** and **B**) Semiquantitative analysis of inflammation extent (based on H&E staining, not shown) (**A**) and (**B**) collagen deposition (based on Masson’s trichrome staining, not shown); *n* = 5–6/group. (**C** and **D**) Decreased expression of *Col1a1* (**C**) and *Fn1* (**D**) tendency in dKO mice. qPCR; *n* = 5–10/group. (**E** and **F**) The number of centrally nucleated fibers (**E**) based on H&E staining and muscle fiber size (**F**) is affected by the lack of dystrophin but not modulated by miR-378 deficiency; *n* = 5–6/group. (**G**) Immunofluorescent staining of eMyHC expression (green) showing its decreased abundance in dKO mice; Hoechst (blue) was used to visualize nuclei. Representative pictures; confocal microscope (LSM-510; Carl Zeiss). Scale bar: 100 μm; *n* = 4–5/group. (**H**) Significant decrease in *Fgf1* mRNA in dKO mice; qPCR; *n* = 7–10/group. (**I** and **J**) The markedly diminished FGF1 protein level in dKO mice as demonstrated by ELISA (**I**) and Western blot (**J**) analysis; *n* = 7–10/group. (**K**) Representative pictures of immunofluorescent staining of slow MyHC isoform (MyHCI, red) and laminin (green) (upper panel) and its calculation, together with histochemical assessment of SDH activity (bottom panel); *n* = 4–5/group. Scale bar: 100 μm. Data are presented as mean ± SEM. *P < 0.05; **P < 0.01; ****P < 0.0001; 1-way ANOVA with Tukey’s post hoc test.

It is commonly known that, in *mdx* mice, the diaphragm muscle is more severely affected than skeletal muscles, which better resemble the DMD pathology (42). Hence, we wanted to evaluate if typical features of the disease that were affected by miR-378 deficiency in hind appendicular muscles might be also recapitulated in the diaphragm of dKO animals. In line with previous results, inflammation was decreased in dKO mice (Figure 8A). Moreover, although collagen deposition was not affected (Figure 8B), the expression of fibrotic markers — *Col1a1* (Figure 8C) and, to a lesser extent, *Fn1* (Figure 8D) **—** was reduced in dKO mice, similarly to the effect observed in gastrocnemius muscle. Concomitantly, no changes in the presence of centrally nucleated fibers (Figure 8E), as well as muscle fiber size (Figure 8F), were observed; however, a decrease in the eMyHC^+^ fibers in dystrophic mice lacking miR-378 was also evident (Figure 8G). Downregulation of FGF1 expression was found in the diaphragm muscle both at the mRNA (Figure 8H) and protein (Figure 8, I and J) levels, suggesting its general role in mediating the effect of miR-378 in dystrophic animals. Finally, we also sought to determine if the expression of slow-twitch fibers, although not changed by the lack of miR-378 in limb muscles, might be affected in the respiratory diaphragm muscle. Though the quantification of MyHCI^+^ fibers did not reveal any alterations, SDH staining clearly showed the abundance of oxidative fibers in *mdx* animals without any apparent changes in mice devoid of miR-378 (Figure 8K).

### The effect of miR-378 deficiency in mdx mice is less pronounced in 6-month-old mice.

**Figure 9:**
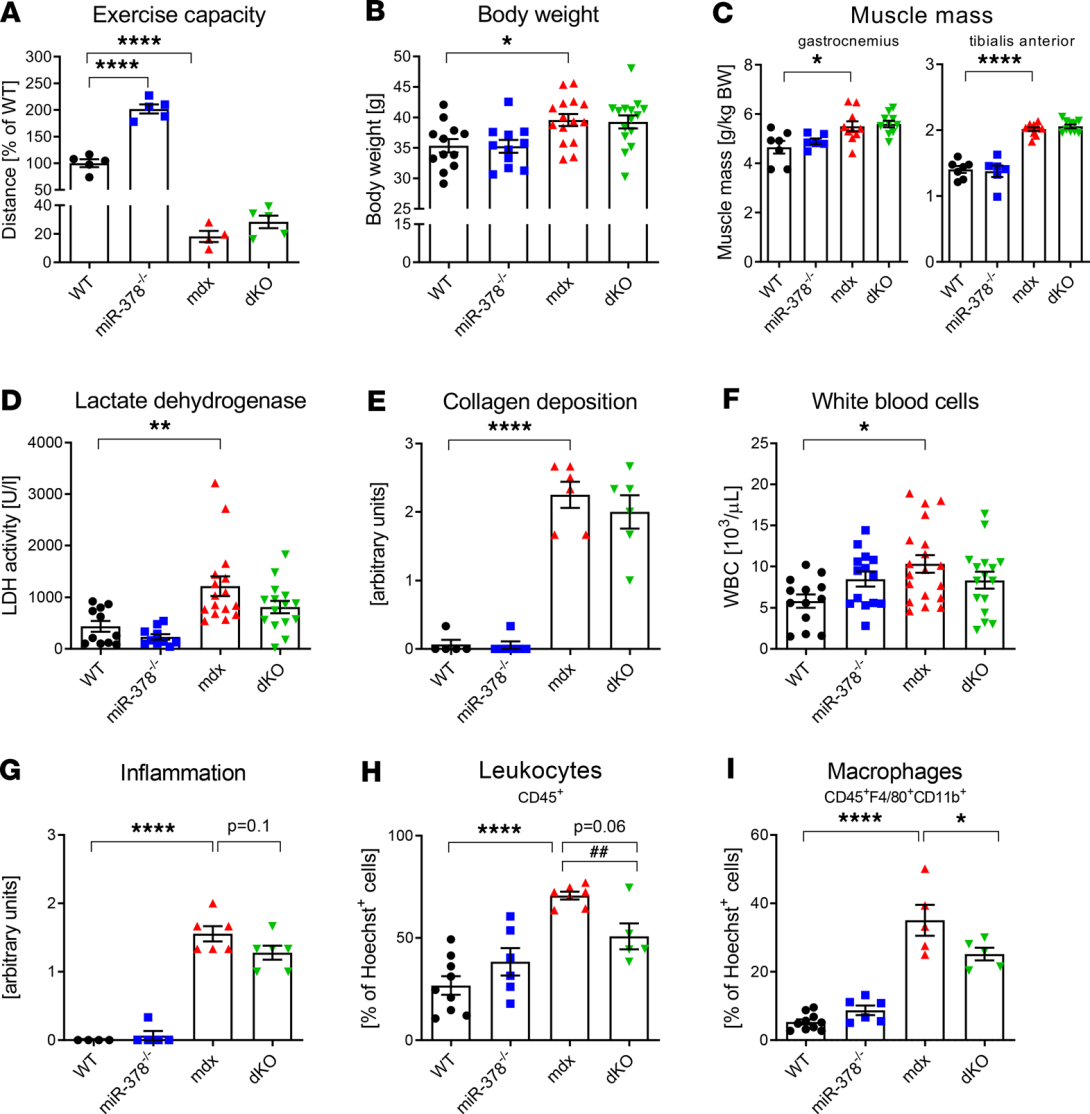
The effect of miR-378 deficiency in 6-month-old animals. (**A**) Muscle performance test indicating the increased running capacity of miR-378^–/–^ animals in comparison with WT mice; downhill running treadmill test presented as the percentage of the running distance to exhaustion compared with WT animals; *n* = 4–5/group. (**B**) Increased body weight of *mdx* animals without the impact of miR-378 loss; *n* = 11–15/group. (**C**) Gastrocnemius and tibialis anterior muscle mass calculated per kg BW showing increased muscle mass in 6-month-old *mdx* mice with no effect of miR-378 deficiency; *n* = 6–10/group. (**D**) Elevated LDH activity in dystrophic animals with no changes in mice lacking miR-378; activity assay; *n* = 10–16/group. (**E**) Semiquantitative analysis of collagen deposition based on Masson’s trichrome staining of the gastrocnemius muscle, indicating no effect of miR-378 loss. Microscopic assessment using Nikon Eclipse microscope; *n* = 5–6/group. (**F**) Increased number of WBC in the peripheral blood; blood cell count; *n* = 13–19/group. (**G**) Semiquantitative analysis of inflammation extent (based on H&E staining) showing increased inflammatory cell infiltration in *mdx* mice with no effect of miR-378 deficiency; microscopic assessment using Nikon Eclipse microscope; *n* = 4–10/group. (**H** and **I**) The analysis of inflammatory cells in hind limb muscles showing the percentage of leukocytes (CD45^+^ cells) (**H**) and macrophages (CD45^+^F4/80^+^CD11b^+^ cells) (**I**); flow cytometry analysis calculated as the percentage of Hoechst^+^ cells; *n* = 5–6/group. Data are presented as mean ± SEM. *P < 0.05; **P < 0.01; ****P < 0.0001 by 1-way ANOVA with Tukey’s post hoc test; ^##^*P* < 0.01 with Student’s *t* test.

To verify if the effect of miR-378 persists over time, we analyzed the phenotype of older, 6-month-old mice. First, we noticed that the exercise capacity of miR-378^–/–^ mice was still higher than WT mice, whereas the physical performance of *mdx* animals was further reduced, with no apparent effect of additional lack of miR-378 (Figure 9A), as compared with 3-month-old animals (Figure 1E). Moreover, no changes in both body weight (Figure 9B) or muscle mass (Figure 9C) were observed due to the miR-378 loss. Similarly, the alleviation of the disease symptoms evidenced by the level of LDH in serum (Figure 9D) and collagen deposition in muscles (Figure 9E) was no longer visible in dKO versus *mdx* animals. Although the number of WBC in the peripheral blood appeared to be increased in *mdx* versus WT counterparts, no further changes as the result of miR-378 deficiency were observed (Figure 9F). Inflammation was still elevated in dystrophic mice, as evidenced by inflammation score in gastrocnemius muscle (Figure 9G) and the number of leukocytes in hind limb muscles (Figure 9H), with the emphasis on macrophages (Figure 9I). Though the effect of miR-378 deficiency was diminished in comparison with younger animals (Figure 2, D–I), a tendency in reduced inflammation (Figure 9G) — together with decreased percentage of leukocytes (Figure 9H) and macrophages (Figure 9I) in hind limb muscles of dKO mice — was still visible, suggesting the involvement of miR-378 loss on inflammation, which, at least to some extent, endure with the increasing age of dystrophic animals.

## Discussion

In our study, we revealed for the first time to our knowledge that the lack of miR-378 attenuates dystrophic phenotype in *mdx* mice. Our initial results demonstrate decreased levels of both mature strands of miR-378 in muscles concomitantly with the elevation of miR-378 in the serum of dystrophic animals, which is in accordance with already published data concerning also DMD patients’ samples (18, 21–23). Moreover, similar regulation of miR-378 in muscles and serum was revealed by us recently in experimental hind limb ischemia, together with the elevated level of miR–378-3p in the plasma of patients with intermittent claudication (17). The differential expression pattern of miR-378 could be explained by the release of miR-378 from the damaged myofibers; however, the active secretion of miR-378 exhibiting paracrine function cannot be ruled out.

One of the major findings of our investigation is that the lack of miR-378 resulted in increased exercise capacity and improved muscle strength of dystrophic animals. This functional improvement is of particular importance, as the progressive character of the disease leading to severe abrogation of physical performance in DMD is recapitulated in dystrophic animals (13, 43). In contrast, Li et al. observed decreased running capability of miR-378–KO animals in comparison with WT counterparts (18). These discrepancies, though they may be related to different experimental procedures, are hard to explain, taking also into account comparable age and sex of animals in both studies. However, the mice used by Li et al. were maintained on a C57BL/6J background, while ours were crossed with *mdx* mice and kept on a mixed background, C57BL/10ScSn × 129SvEv/C57BL/6. Furthermore, the involvement of miR-378 deficiency, specifically in dystrophic animals, was not addressed in that study (18).

We initially suspected that the increased running capability of dystrophic animals devoid of miR-378 will be reflected by the changes in the composition of different muscle fiber types, as it is commonly known that the augmented abundance of so-called type I, slow-twitch, or oxidative fibers improves long-distance running (38). Surprisingly, in hind limb muscles, the percentage of slow-twitch fibers was elevated already in severely affected *mdx* mice, without their further escalation driven by the lack of miR-378. This might indicate different regulation of muscle fiber type transition upon healthy and diseased conditions. Indeed, in accordance with our results, an increased oxidative fiber content was reported in both mouse (44, 45) and canine models (46) of DMD, as well as in DMD patients (47). Concomitantly, it was demonstrated that type II, glycolytic fibers are prone to damage and degenerate first, whereas type I fibers are relatively spared (47). In line with that, our data show a profound decrease in the percentage of oxidative fibers at the expense of glycolytic fibers in dKO animals in tibialis anterior muscle, resembling the situation found in WT animals and suggesting lower degeneration of muscle fibers as the result of miR-378 deficiency. Though the level of tissue damage markers in serum, such as LDH activity, was decreased in dKO animals, we did not observe any apparent differences in the necrosis appearance. Nonetheless, further studies are warranted, especially because the role of miR-378 in skeletal muscle apoptosis and autophagy was also proposed recently (18).

We also undermined the possible impact of miR-378 on vascularization under dystrophic conditions. Nonetheless, similar to our recent findings, the muscle VEGFA level in dystrophic animals was diminished (48), which was accompanied by the reduced expression of other angiogenesis-related genes such as *Pecam1*, further indicating possible alterations in the angiogenesis process in *mdx* mice (49).

In accordance with Carrer et al., we did not notice significant changes in the body weight and muscle mass in miR-378^–/–^ mice (50); however, additional lack of miR-378 in dystrophic animals reduced their weight. It has to be noted that both true muscle hypertrophy and so-called pseudohypertrophy attributed mostly to the accumulation of fat and connective tissue are the characteristic features of DMD patients recapitulated also in *mdx* mice (51). Nonetheless, as muscle fiber size was not altered by the lack of miR-378, we rather hypothesized that decreased muscle mass of dystrophic animals devoid of miR-378 might be due to the diminished inflammation and fibrosis extent. Such an assumption was also supported by the mitigated expression of HO-1 previously shown by us to be highly expressed by immune cells abundantly infiltrating muscles of dystrophic animals (13).

Here, we provide evidence that the lack of miR-378 prominently reduces muscle inflammation, a hallmark of DMD pathology. Recently, Zhang et al. demonstrated that miR-378 promotes hepatic inflammation and fibrosis (52), and Jiang et al. observed increased miR-378 expression upon stimulation with IL-6, TNF-α, and leptin in human adipocytes (53). Interestingly, miR–378-3p upregulation was reported in macrophages upon IL-4 stimulation (54), and we further demonstrate that miR-378 loss diminished the abundance of macrophages, as well their M1-like and M2-like subpopulations in dystrophic animals. Macrophage subpopulations are essential mediators of muscle regeneration, with proinflammatory M1-like macrophages stimulating proliferation of myogenic cells and M2-like macrophages restricting cytolytic damage, increasing differentiation of mSCs and myofiber growth (26). Both subpopulations of macrophages coexist in the muscles of dystrophic animals, orchestrating the inflammation process. However, apart from that, M2-like macrophages were also linked to fibrosis (55).

The impact of miR-378 on fibrosis seems to be complex and tissue dependent. The antifibrotic role of miR-378 was demonstrated in the heart (56), whereas, in the liver, both antifibrotic (57) and profibrotic (52) effects of miR-378 were reported. Nonetheless, to the best of our knowledge, no studies evaluated the role of miR-378 on fibrosis, particularly in the skeletal muscles of dystrophic animals. In our hands, inhibition of miR-378 resulted in diminished fibrosis, predominantly in gastrocnemius muscle, together with the decreased expression of fibrotic markers, namely *Col1a1* and *Fn1*. Our results are further strengthened by the fact that the abundance of FAPs, which contribute significantly to the fibrosis and fat deposition in dystrophic animals (31), was markedly diminished in *mdx* mice lacking miR-378. This could be, at least partially, explained by the decreased percentage of eosinophils, which were shown to activate resident FAPs by the release of IL-4 (58). Moreover, eosinophil-derived major basic protein-1 (MBP-1) promotes fibrosis in skeletal and cardiac muscles of dystrophic animals (28). Our unpublished data indicate decreased MBP-1 levels in the liver of dKO versus *mdx* animals, providing a possible link between reduced fibrosis, FAPs abundance, and immune response driven by the lack of miR-378.

In the current study, we revealed alterations in mSCs, the key players in muscle regeneration (6). So far, the literature data regarding the function of miR-378 in mSCs are rather inconclusive. Although in vitro studies performed on C2C12 myoblasts demonstrated that miR-378 accelerates differentiation by targeting either musculin, the myogenic differentiation inhibitor (20), or bone morphogenetic protein 4 (BMP4) (59), in vivo experiments on primary mSCs revealed that Tg mice overexpressing miR-378 exhibit delayed muscle regeneration upon acute, cardiotoxin-induced muscle injury by direct targeting insulin-like growth factor 1 receptor (*Igf1r*) (24). In the current study, the KO of miR-378 in dystrophic animals did not alter the number of mSCs but resulted in a diminished contribution of activated mSCs within the total mSCs, together with decreased proliferation status of mSCs. Furthermore, ex vivo analyses revealed decreased fusion of mSCs, together with morphological changes of differentiated myotubes. Though obtained data cannot be directly translated to in vivo studies, it might be proposed — but warrants further verification — that alterations of mSC properties are due to the lower inflammation and fibrosis in *mdx* mice lacking miR-378. In line with that, the abundance of newly formed, eMyHC^+^ fibers was consistently decreased in all analyzed muscles of dKO animals, though no differences in the presence of centrally nucleated fibers and overall muscle fiber size were noticed. It has to be underlined that there is no single, explicit marker of the regeneration process. The presence of centronucleated fibers, together with the morphological changes in the size of muscle fibers, has served as the most commonly used indicator of muscle regeneration for years; however, the limitations and variability of this marker were also stressed (35). Alternatively, eMyHC has been suggested as the robust biomarker of the regeneration process (60). In the latter study, the restoration of dystrophin or utrophin overexpression reduced the level of eMyHC, accompanied by the rescued muscle function of dystrophic mice (60).

The understanding of the direct mechanism that could be responsible for all of the phenotypic changes observed by us in dystrophic animals lacking miR-378 becomes challenging. It has to be emphasized that mice used in this study are devoid of both forms of miR-378 — namely, miR–378-3p and miR–378-5p, which possess divergent “seed” regions; thereby, they are capable to regulate mRNA of different targets. Here, we demonstrate FGF1 as one of the possible mediators of changes driven by the lack of miR-378. In accordance with this data, we have recently revealed decreased expression of FGF1 in the C2C12 myoblast cells in which miR-378 was silenced (17). Strikingly, according to the miRWalk database, FGF1 is one of the predicted — yet not validated — targets of miR–378-5p, suggesting that decreased FGF1 is due to indirect regulation of FGF1 by miR-378. FGF1 is a member of the heparin-binding growth factor family with well-established mitogenic properties (61). In muscle biology, it is considered as an inhibitor of muscle differentiation (62), but some reports emphasize the opposite (63), with the differential effects of extracellular and intracellular FGF1 (64). Apart from that, the involvement of FGF1 in the fibrosis was also demonstrated (65). More in-depth studies are warranted to fully understand the role of FGF1 under dystrophic conditions. Particularly, it would be of great interest to evaluate the effect of FGF1 inhibitors on dystrophic phenotype.

Importantly, some findings of our study extend beyond the typical, muscle-specific symptoms of the disease. An increased testosterone level in the serum of dystrophic mice lacking miR-378 could be, at least partially, responsible for improved performance of dKO mice. In line with that, androgen receptor modulators were shown to enhance muscle performance (66), extend survival, and improve cardiopulmonary functions in *mdx* mice (67).

Altogether, the results obtained by us strongly suggest that targeting miR-378 may provide an approach to modulate the severity of the disease. Nonetheless, it has to be noted that, though the improved running capacity of miR-378^–/–^ mice was still apparent in older animals, beneficial effects in dystrophic mice lacking miR-378 declined with age. The significance of that has to be further investigated. In conclusion, we propose that the investigation of agents modulating miR-378 level could help in combined therapies for DMD patients (68).

## Methods

### Animal models.

*Mdx* mice C57BL/10ScSn-*Dmd^mdx^*/J and control mice, C57BL/10ScSnJ, were purchased from the Jackson Laboratory (stock nos. 001801 and 000476, respectively). miR-378^–/–^ mice at 129SvEv/C57BL/6 background were the gift of Eric Olson (Department of Molecular Biology, University of Texas Southwestern Medical Center, Dallas, Texas, USA) (50). To generate dKO mice globally lacking both dystrophin and miR-378, *Dmd^mdx/mdx^* female mice were crossed with miR-378^–/–^ male mice to generate *Dmd^mdx/+^*miR-378^+/–^ females or *Dmd^mdx/Y^*miR-378^+/–^ offsprings, which were bred together to obtain male dKO *Dmd*^mdx/Y^**miR-378^–/–^ mice at mixed background C57BL/10ScSn × 129SvEv/C57BL/6. *Dmd^+/Y^*miR-378^+/+^ (WT), *Dmd*^mdx/Y^**miR-378^+/+^ (*mdx*), and *Dmd^+/Y^*miR-378^–/–^ (miR-378^–/–^) animals used for the experiments were also bred at mixed background C57BL/10ScSn × 129SvEv/C57BL/6. Only 3- and 6-month-old male littermates or age-matched mice (from generations F3 to F6) were used for experiments.

### Muscle performance and contractile measurements.

The treadmill exhaustion test was performed by the investigator blind to the mice genotype using the protocol described previously (13). The absolute maximum force of the tibialis anterior muscle was determined in situ using Aurora 1300A: 3-in-1 Whole Animal System (Aurora Scientific) according to other studies, with the pulse frequency of 1–125 Hz (69, 70).

### Blood cell count.

The blood from mice was collected from vena cava directly to EDTA-coated tubes and was analyzed by the ABC Vet instrument (Horiba ABX). The patients’ samples were obtained and analyzed on a routine basis from 25 DMD boys (3–7 years old) before glucocorticoid treatment at the Department of Neurology, Medical University of Warsaw, Poland.

### Histological and immunofluorescent analysis of the muscles.

For histological analyses, muscles were either embedded in OCT (Leica) and frozen in liquid nitrogen–cooled isopentane or fixed for 48 hours in 10% formalin, and paraffin embedded. A total of 8 μm– to 10 μm–thick frozen sections was prepared, air-dried for at least 2 hours, and kept at –20°C for further H&E staining, the analysis of regeneration extent (assessed based on the presence of centrally nucleated myofibers), and the evaluation of the muscle fiber’s size and necrosis. Paraffin sections (4-μm thick) were deparaffinized and subjected to Masson’s trichrome staining. All of the above procedures were performed according to our recent studies (13, 48, 71).

For NADH dehydrogenase or SDH activity assessment indicating the extent of oxidative fibers, the sections were air dried for 15 minutes before the staining. In the case of NADH dehydrogenase activity determination, the slides were incubated at 37°C for 30 minutes in a reaction mixture (1:1) prepared from 8 mg/5 mL NADH and 10 mg/5 mL nitro blue tetrazolium chloride (NBT). For SDH activity assessment, the slides were incubated at 37°C for 60 minutes in 0.2M sodium succinate solution in phosphate buffer containing 1 mg/mL NBT. Next, the slides were washed 3 times in deionized water, and the unbound staining was removed by the increasing concentration of acetone (30%, 60%, 90%). All reagents were purchased from MilliporeSigma. Finally, the slides were washed several times in deionized water and mounted in a Histofluid (Marienfeld) medium. Based on the staining results, the muscle fibers were assigned as pale (glycolytic) or dark (oxidative).

Immunofluorescent staining of different muscle fiber types was performed as described by Dyar et al. (72). Primary antibodies against MyHC isoforms (all from Developmental Studies Hybridoma Bank [DSHB]; mouse-specific to MyHCI [BA-D5, IgG2b, supernatant], MyHCIIa [SC-71, IgG1, supernatant], and eMyHC [F1.652, IgG1, supernatant]), as well as rat-specific to laminin (ab11576, Abcam) to visualize individual muscle fiber, were used following incubation with secondary antibodies (all from Thermo Fisher Scientific). Secondary antibodies include goat anti–mouse IgG2b Alexa Fluor 568 (A-21144) for detection of MyHCI (in red) and/or goat anti–mouse IgG1 Alexa Fluor 488 (A-21121) for detection of MyHCIIa/eMyHC (in green), as well as goat anti–rat IgG AlexaFluor488 (A-11006) for detection of laminin (in green).

Immunofluorescent staining of Pax7 or CD31/α-SMA was performed on the gastrocnemius frozen sections as described previously (17, 48). The stainings were visualized under Nikon Eclipse Ti microscope or Zeiss LSM-880 meta laser scanning confocal microscope and analyzed by the investigator blind to the mice genotype using ImageJ software (NIH). If necessary, the brightness and/or contrast was adjusted to all of the pictures equally.

### Serum CK and LDH measurement.

Serum was obtained by blood collection from vena cava just before the terminal procedure and muscle collection by allowing the blood to clot at room temperature for 30 minutes and then centrifuged at 1000 *g* for 10 minutes. The activity of CK and LDH was measured using the diagnostic Liquick Cor-CK and Liquick Cor-LDH kits, respectively (Cormay) according to the vendor’s instruction.

### ELISA tests.

FGF1/VEGFA content in muscles and serum testosterone levels were determined followed by the vendor’s instructions (R&D Systems and Abcam, respectively).

### Western blotting.

After the mice were sacrificed, tissues were immediately collected, snap-frozen in liquid nitrogen, and stored at –80°C for downstream analyses. Muscles were lysed in 200–350 μL ice-cold 1% Triton X100 in PBS containing proteinase inhibitors (Roche Diagnostic), homogenized using TissueLyser (QIAGEN), and 25 μg of protein lysate was processed as described previously (13). Primary antibodies — mouse monoclonal anti-FGF1 (AF4686, R&D Systems), mouse monoclonal anti-GAPDH (sc-59540, Santa Cruz Biotechnology Inc.), rabbit polyclonal anti–HO-1 (ADI-SPA-894, Enzo Life Sciences), rabbit polyclonal anti-AMPKA (2603S, Cell Signalling Technology), and rabbit monoclonal anti–PGC1-β (ab176328, Abcam) — and secondary antibodies (conjugated with HRP) include anti–mouse Ig (554002, BD Biosciences) for the detection of GAPDH or FGF1, as well as anti–rabbit IgG (7074, Cell Signaling Technology) for the detection of HO-1, AMPKA, PGC1-β, were used.

### Flow cytometry analysis of mononucleated cell populations in skeletal muscles.

The preparation of cells for flow cytometry analysis was performed as described previously (13, 48, 71). For the analysis of leukocytes, macrophages and eosinophils populations, the following antibodies were used: rat anti–mouse CD45-APC–eFluor780 (1:30, 30-F11), rat anti–mouse CD86–PE (GL1), and rat anti–mouse F4/80–APC (BM8) (all from Thermo Fisher Scientific Waltham); rat anti–mouse MHCII–PE-Cy7 (M5/114.15.2) and rat anti–mouse CD11b–PE (M1/70) (from BD Biosciences); and rat anti–mouse CD206–PerCP/Cy5.5 (C0682C2, BioLegend). Antibodies for the analysis of mSCs included the following: rat anti–mouse α7 integrin–PE (a7i, 334908, R&D Systems), rat anti–mouse CD34–Alexa Fluor 700 (RAM34), and rat anti–mouse CD45–APC-eFluor780 (30-F11) (Thermo Fisher Scientific), as well as rat anti–mouse CD31–APC (MEC 13.3) and rat anti–mouse Sca1–PE-Cy7 (D7) (from BD Biosciences). Proliferating mSCs were determined based on Hoechst 33342 (MilliporeSigma) staining. The stained cells were analyzed using LSRFortessa flow cytometer with FACSDiva (BD Biosciences).

### Isolation and differentiation of mSCs.

mSC isolation and culture were done accordingly to our previous study (13, 48). To induce differentiation 24 hours after isolation, cells were switched to differentiation medium (DMEM with 2% horse serum) for 3 days. Afterward, cells were stained with primary mouse anti-MyHC antibody (M4276, MilliporeSigma) followed by the incubation with goat anti–mouse IgG AlexaFluor488 secondary antibody (A28175, Thermo Fisher Scientific) according to the protocol described previously (13). Cells were analyzed under fluorescent microscope Nikon Eclipse T*i*. Quantification of the fusion index was determined by the percentage of MyHC^+^ fibers that contained 3 or more nuclei among the total number of nuclei.

### RNA isolation, reverse transcription PCR (RT-PCR) and quantitative PCR (qPCR).

After the mice were sacrificed, muscles were collected in tubes containing RNAlater Stabilization Solution (MilliporeSigma), immediately snap-frozen in liquid nitrogen, and stored at –80°C for downstream analyses. Total RNA was isolated using the standard Chomczynski-Sacchi method (73). The concentration and quality of RNA were determined by NanoDrop Spectrophotometer (Thermo Fisher Scientific). RT-PCR was performed as described previously (13) or according to the vendor’s instruction using miRCURY LNA RT Kit (QIAGEN) in the case of miRNAs expression analysis.

qPCR was performed using StepOne Plus Real-Time PCR (Applied Biosystems, Thermo Fisher Scientific) with 10 ng of cDNA, SYBR Green PCR Master Mix (MilliporeSigma), and specific primers (listed in Supplemental Table 4). *Eef2* was used as a housekeeping gene. LNA miRCURY RT-PCR Kit (QIAGEN) was used for miRNA determination. Specific miRNA expression (Supplemental Table 5) was normalized to the constitutive small RNA U6 or miR-16 (geometric average of miR–16-3p and miR–16-5p) in the case of determination of serum miRNA level using miRNeasy Serum/Plasma Kit (QIAGEN) (74). Relative quantification of gene expression was calculated based on the 2^–ΔCt^ method (ΔC_t_ = C_t gene of interest_ – C_t housekeeping gene_) and presented as the relative expression in comparison to WT animals.

### RNA sequencing analysis.

For RNA sequencing analysis, gastrocnemius muscles from 3-month-old mice were preserved in RNAlater Stabilization Solution (MilliporeSigma) and immediately snap-frozen in liquid nitrogen. After RNA isolation, the total RNA quality was analyzed with the RNA 6000 Nano Kit on Agilent 2100 Bioanalyzer (Agilent). Only samples with an RNA integrity number (RIN) > 7 were considered for downstream analyses.

Messenger RNA (poly[A]-containing mRNA) was purified from the 1–8 μg of total RNA by magnetic beads coated with oligo(dT) using a Dynabeads mRNA DIRECT Micro Kit (Thermo Fisher Scientific). To estimate the quality and quantity of mRNA samples, we analyzed samples with the RNA 6000 Pico Kit with the 2100 Bioanalyzer (Agilent). The sequencing library of each RNA sample was prepared by using Ion Total RNA-Seq Kit v2 according to the manufacturer’s protocol (Thermo Fisher Scientific). The libraries were prepared from 1–15 ng of mRNA. Briefly, the mRNA was fragmented using RNAseIII and then purified. The fragmented RNA was hybridized and ligated with Ion adaptors. The RNA fragments were then reverse transcribed and amplified to double-stranded cDNA. Next, the cDNA was purified by the magnetic bead–based method. The molar concentration and size selection (50–1000 bp) of each cDNA library were determined using DNA 1000 Kit on Bioanalyzer 2100 (Agilent).

Each library was diluted to ~80 pM concentration before template preparation, and up to 4 barcoded libraries were mixed in equal volume and used for automatic template preparation on Ion Chef (Thermo Fisher Scientific) instrument using reagents from the Ion PI Hi-Q Sequencing 200 Kit and Ion PI v3 Proton Chip. Samples were sequenced on the Ion Proton System according to the manufacturer’s instructions (Thermo Fisher Scientific).

Raw reads were mapped to mm10 reference genome with Ion Torrent RNASeq Analysis Plugin version 5.0, which uses STAR (75) as the primary aligner, and bowtie2 for previously unaligned reads (76). Gene abundance was quantified with htseq-count (HTSeq framework version 0.6) (77) using Ensembl Gene gtf file from University of California Santa Cruz (Santa Cruz, California, USA) as a reference. Differential gene expression was performed with R package DESeq2 version 1.10.1. Overrepresentation of KEGG pathways in genes that were differentially expressed was performed with R package ClusterProfiler version 3.6 (78) and visualized with pathview version 1.18 (79). Data were deposited in the BioProject database (ID PRJNA591429).

### Statistics.

Data are presented as mean ± SEM. Differences between groups were tested for statistical significance using the 1-way or 2-way ANOVA, followed by Tukey’s post hoc test or, when indicated, the unpaired 2-tailed Student’s *t* test. *P* < 0.05 was considered significant, and *P* ≤ 0.1 was marked as a tendency. The outliers were identified based on the Grubb’s test.

### Study approval.

All animal procedures and experiments were performed in accordance with national and European legislation, after approval by the first or second Local Institutional Animal Care and Use Committee in Kraków, Poland (approval numbers 66/2013, 322/2018 and 191/2019). Animals were kept in specific pathogen–free (SPF) conditions with water and food available ad libitum. Patients’ blood parameters were collected retrospectively and were based on the routine analyses done for justified and necessary medical diagnostic procedures that do not require bioethical approval.

## Author contributions

JD and AŁ designed the study; PP, OM, IBB, M. Kozakowska, KPG, AC, UGK, KBS, MC, M. Kulecka, and AŁ performed the experiments; PP, AŁ, and JD wrote the main manuscript text; PP, AŁ, and OM prepared the figures; APC and AKP collected the patients’ data; and AJ, AŁ, JD, M. Kozakowska, JO, and MM supervised the research. All authors approved and commented on the manuscript.

## Supplementary Material

Supplemental data

## Version 1 06/04/2020

Electronic publication
