# Supplementary material for: Lack of miR-378 attenuates muscular dystrophy in mdx mice
Source: JCI Insight. 2020 Jun 4;5(11):e135576. doi: 10.1172/jci.insight.135576 (PMC7308053; doi:10.1172/jci.insight.135576)
Supplement: Supplemental data [file jciinsight-5-135576-s050.pdf]

Table S1. The blood cell count in 3-7 years old DMD patients (n=25) before glucocorticoid therapy indicating no severe abnormalities in the level of white blood cells.

Parameter	***Average ± SEM***	**Norm**
White blood cell count (WBC), x 10^3/µl	8.3 ± 0.3	(4.0 – 11.0)
Neutrophils, x 10^3/µl	3.7 ± 0.3	(1.9 – 8.0)
Lymphocytes, x 10^3/µl	3.6 ± 0.2	(0.9 – 5.2)
Monocytes, x 10^3/µl	0.54 ± 0.03	(0.16 – 1.00)
Eosinophils, x 10^3/µl	0.3 ± 0.1	(0.0 – 0.8)
Basophils, x 10^3/µl	0.033 ± 0.005	(0.000 – 0.200)
Neutrophils, % WBC	↓ 44.2 ± 2.3	(50.0 – 66.0)
Lymphocytes, % WBC	↑ 44.9 ± 2.1	(20.0 – 40.0)
Monocytes, % WBC	6.6 ± 0.4	(4.0 – 8.0)
Eosinophils, % WBC	3.7 ± 0.6	(2.0 – 4.0)
Basophils, % WBC	0.5 ± 0.1	(0.0 – 1.0)

Table S2. RNA sequencing analysis results showing genes downregulated (green) and upregulated (red) in miR-378$^{-/-}$ mice in comparison to the WT mice that are changed at least 1.5 times with p_{adj}<0.1. Segregation was done based on the fold change values.

Gene symbol	Gene full name	Fold change	p_{adj}
	Immune system		
Btla	B- and T-lymphocyte attenuator	7.41	0.0078
Iigp1	Interferon-inducible GTPase 1	3.66	0.00020
Ly96	lymphocyte antigen 96	0.22	0.090
	Fibrosis and ECM		
Col6a6	collagen type VI alpha 6 chain	2.88	0.0054
Fgf1	fibroblast growth factor 1	0.19	5.09E-15
	Metabolism		
Blvrb	biliverdin reductase	2.86	0.028
Rpusd4	RNA pseudouridylate synthase domain containing 4	0.65	0.090
St6galnac4	ST6 N-acetylgalactosaminide alpha-2,6-sialyltransferase 4	0.43	0.062
Gnpda1	glucosamine-6-phosphate deaminase 1	0.25	0.0017
Aldh1b1	aldehyde dehydrogenase 1 family, member B1	0.11	0.098
B3gat1	beta-1,3-glucuronyltransferase 1	0.06	0.083
	Genetic Information Processing and Regulation		
Dbp	D site albumin promoter binding protein	3.48	0.012
Egr1	early growth response 1	3.45	0.073
Rpl34	ribosomal protein L34	3.26	0.0069
Klhl38	kelch-like 38	3.20	0.0022
Rplp0	ribosomal protein, large, P0	1.86	0.090
Rnps1	ribonucleic acid binding protein S1	1.81	0.081
Urm1	ubiquitin related modifier 1	0.66	0.069
Noct	nocturnin	0.44	0.091
Snora34	small nucleolar RNA, H/ACA box 34	0.30	0.027
G0s2	G0/G1 switch gene 2	0.30	0.041
Rpl26	ribosomal protein L26	0.08	2.5E-05
	Signal transduction		
Map3k8	mitogen-activated protein kinase 8	2.86	0.0039
Pik3ip1	phosphoinositide-3-kinase interacting protein 1	1.97	0.022
Fbxo32	F-box protein 32	1.79	0.028
	Muscles		
Tnnt1	troponin T1, skeletal, slow	2.94	0.027
Myh7b	myosin, heavy chain 7B, cardiac muscle, beta	2.83	0.078
Atp2a2	ATPase, Ca++ transporting, cardiac muscle, slow twitch 2	2.34	0.097
Myod1	myogenic differentiation 1	0.56	0.027
Myhas	myosin heavy chain gene antisense RNA	0.29	0.039
	Nervous system		
Ngef	neuronal guanine nucleotide exchange factor	13.70	0.091
Qrfp	pyroglutamylated RFamide peptide	5.18	0.0017
Syt12	synaptotagmin XII	0.35	0.074
Pak3	p21 protein (Cdc42/Rac)-activated kinase 3	0.03	0.052
	Heat shock protein		
Hsph1	heat shock 105kDa/110kDa protein 1	0.58	0.027
Hsp90aa1	heat shock protein 90, alpha (cytosolic), class A member 1	0.29	0.042
Hspa1b	heat shock protein 1B	0.21	0.064
Hspa1a	heat shock protein 1A	0.17	0.0048
	Other		
Dlk1	delta like non-canonical Notch ligand 1	5.82	0.0046
Adamts20	a disintegrin-like and metallopeptidase (reprolysin type) with thrombospondin type 1 motif, 20	3.05	0.0017
Gadd45b	growth arrest and DNA-damage-inducible 45 beta	3.02	0.0017
Nr1d1	nuclear receptor subfamily 1, group D, member 1	2.27	0.00052
Slc47a1	solute carrier family 47, member 1	0.60	0.0093
Mrm1	mitochondrial rRNA methyltransferase 1	0.54	0.097
Rsph1	radial spoke head 1 homolog	0.41	0.014
Abra	actin-binding Rho activating protein	0.36	0.0015
Slc37a1	solute carrier family 37, member 1	0.11	0.0017
Mir133b	microRNA 133b	0.04	0.033

Table S3. RNA sequencing analysis results showing genes downregulated (green) and upregulated (red) in dKO mice in comparison to the mdx mice that are changed at least 1.5 times with p_{adj}<0.1. Segregation was done based on the fold change values.

Gene symbol	Gene full name	Fold change	p_{adj}
Immune system			
H2-Q10	histocompatibility 2, Q region locus 10	9.93	5.7E-15
Iigp1	Interferon-inducible GTPase 1	4.46	7.8E-07
H2-K1	histocompatibility 2, K region locus 2	0.50	0.058
C1qc	complement component 1, q subcomponent, C chain	0.46	0.069
Mgl2	macrophage galactose N-acetyl-galactosamine specific lectin 2	0.44	1.95E-05
C1qb	complement C1q subcomponent subunit B	0.36	0.0065
H2-D1	histocompatibility 2, D region locus 1	0.14	5.56E-44
H2-T-ps	histocompatibility 2, T region locus, pseudogene	0.09	2.79E-06
H2-Q5	histocompatibility 2, Q region locus 5	0.06	0.0090
H2-Q7	histocompatibility 2, Q region locus 7	0.05	1.36E-10
H2-Ea-ps	histocompatibility 2, class II antigen E alpha, pseudogene	0.01	4.52E-05
Fibrosis and ECM			
Nbl1	neuroblastoma suppressor of tumorigenicity 1	0.57	0.042
Spon2	spondin 2	0.44	0.0052
Fgf1	fibroblast growth factor 1	0.20	3.17E-15
Metabolism			
Eno1b	enolase 1B	48.40	2.79E-06
Tnxa	tenascin XA	14.40	0.018
Aldh1a1	aldehyde dehydrogenase family 1, subfamily A1	1.96	0.098
St8sia5	ST8 alpha-N-acetyl-neuraminide alpha-2,8-sialyltransferase 5	1.60	0.041
Galnt16	polypeptide N-acetylgalactosaminyltransferase 16	0.62	0.030
Cox18	cytochrome c oxidase assembly protein 18	0.47	0.052
Gnpda1	glucosamine-6-phosphate deaminase 1	0.38	0.0092
Hddc3	HD domain containing 3	0.34	0.028
Genetic Information Processing and Regulation			
Nr4a3	nuclear receptor subfamily 4, group A, member 3	5.02	0.074
Igfn1	immunoglobulin-like and fibronectin type III domain containing 1	2.37	0.098
Rps18	ribosomal protein S18	0.31	0.029
Rpl26	ribosomal protein L26	0.08	1.53E-05
Rplp0-ps1	ribosomal protein, large, P0, pseudogene 1	0.02	0.0077
Signal transduction			
Sfrp2	secreted frizzled-related protein 2	0.31	4.52E-05
Other			
Fam20c	family with sequence similarity 20, member C	0.58	0.030
Pex6	peroxisomal biogenesis factor 6	0.57	0.097
Tuba1c	tubulin, alpha 1C	0.39	0.00071
Cdkn2a	cyclin-dependent kinase inhibitor 2A	0.32	0.056

Table S4. The sequences of forward (F) and reverse (R) primers used for the determination of gene expression on mRNA level by qRT-PCR.

Gene	***Sequence 5' → 3'***
Ampka1	*F: TGTGACAAGCACATTTTCCAA*
	R: CCGATCTCTGTGGAGTAGCA
Ampka2	*F: ACAGCGCCATGCATATTCCT*
	R: TCCGACTGTCTACCAGGTAA
Col1a1	*F: CGATCCAGTACTCTCCGCTCTTCC*
	R: ACTACCGGGCCGATGATGCTAACG
Eef2	*F: AGAACATATTATTGCTGGCG*
	R: CAACAGGGTCAGATTTCTTG
Essra	*F: AGAGCCAGCCAGTCCTGACA*
	R: TCACAGGATGCCACACCGTA
Essrg	*F: GGAAGAATTCGTCACCCTCA*
	R: TTCTGCACAGCTTCCACATC
Fgf1	*F: ATGGACACCGAAGGGCTTTT*
	R: GAGGCCCACAAACCAGTTCT
Fn1	*F: AGCCTGCTCATCAGTTGGGA*
	R: GATGGAAACTGGCTTGCTGC
Pecam1	*F: CCAAAGCCAGTAGCATCATGGTC*
	R: GGATGGTGAAGTTGGCTACAGG
Ppara	*F: ACTACGGAGTTCACGCATGTG*
	R: TTGTCGTACACCAGCTTCAGC
Ppard	*F: GCCCAAGTTCGAGTTTGCTGT*
	R: ATTCTAGAGCCCGCAGAATG
Ppargc1a	*F: TTGACTGGCGTCATTCGGGA*
	R: GCAGGCTCATTGTTGTACTG
Ppargc1b	*F: CTGACACGCAGGGTGGG*
	R: AGGTCAAGCTCTGGCAAGTC

Table S5. The sequences of primers used for the determination of miRNA level using miRCURY LNA RT-PCR Kit.

miRNA	Sequence
miR-378-3p	*5'-ACUGGACUUGGAGUCAGAAGG-3'*
miR-378-5p	*5'-CUCCUGACUCCAGGUCCUGUGU-3'*
miR-16-3p	*5'-CCAGUAUUGACUGUGCUGCUGA-3'*
miR-16-5p	*5'-UAGCAGCACGUAAAUAUUGGCG-3'*
U6 snRNA	*5'-CGCAAGGATGACACGCAAATTC-3'*

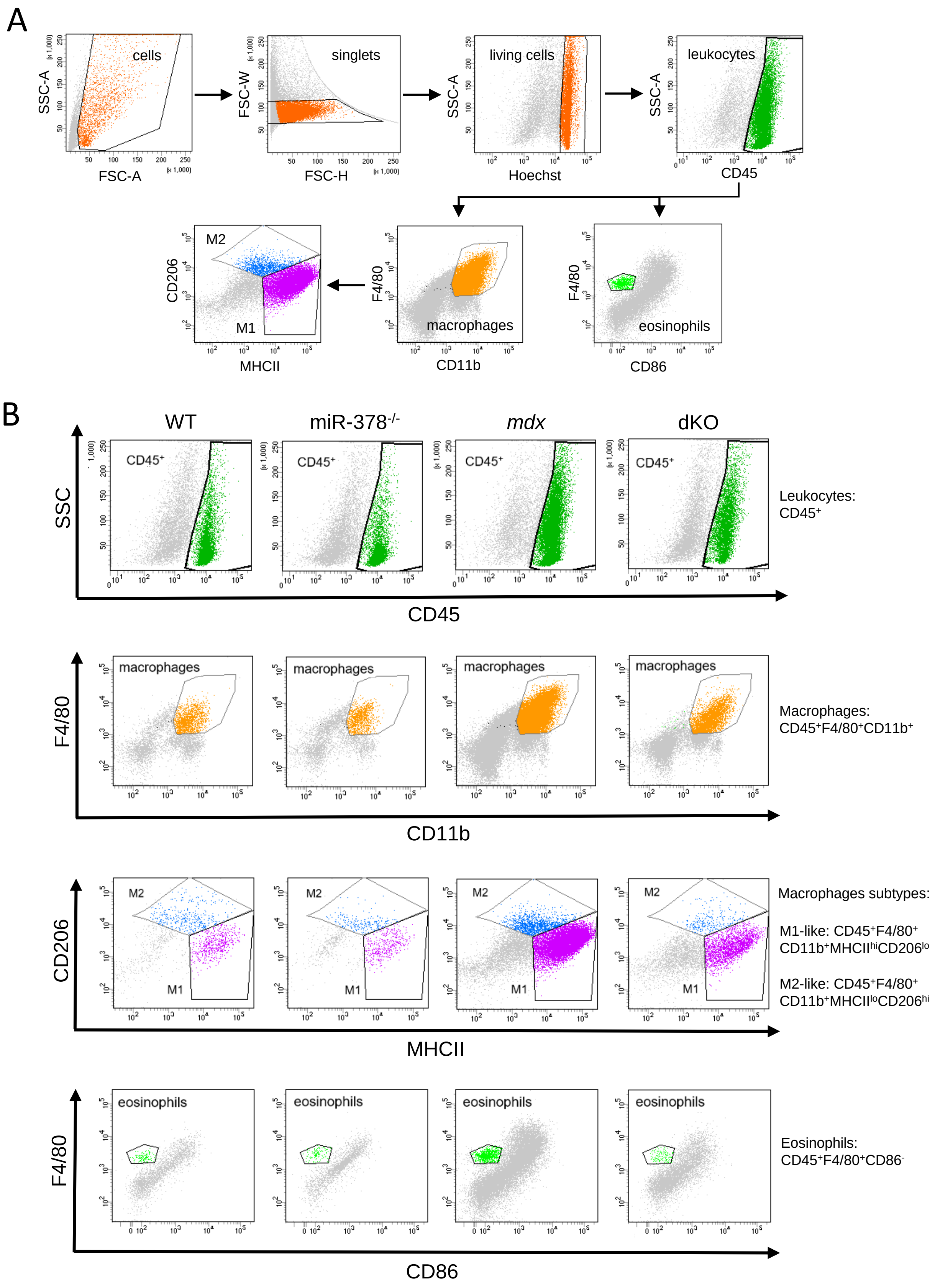

Supplementary Figure 1. The analysis of immune cells populations within skeletal muscles of hind limbs of WT, miR-378$^{-/-}$, *mdx* and dKO mice based on flow cytometry analysis. (A) Gating strategy. **(B)** Examples of two-parameter flow cytometry dot plots for discrimination of leukocytes (CD45^{+} cells), macrophages (CD45^{+}F4/80^{+}CD11b^{+} cells), M1-like macrophages (CD45^{+}F4/80^{+}CD11b^{+}MHCII^{hi}CD206^{lo}), M2-like macrophages (CD45^{+}F4/80^{+}CD11b^{+}MHCII^{lo}CD206^{hi}) and eosinophils (CD45^{+}F4/80^{+}CD86^{-}).

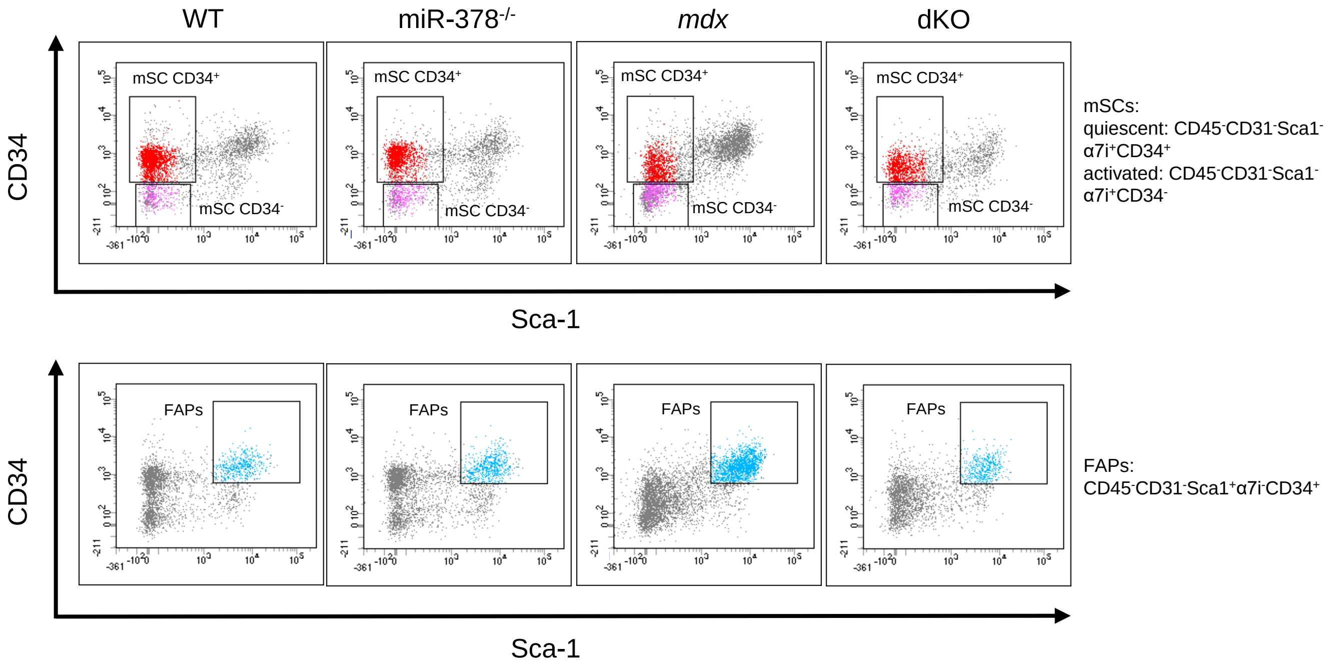

Supplementary Figure 2. The analysis of mSCs and FAPs populations within skeletal muscles of hind limbs of WT, miR-378$^{-/-}$, *mdx* and dKO mice. Examples of two-parameter flow cytometry dot plots for discrimination of quiescent (CD45^{-}CD31^{-}Sca1^{-} α7i^{+}CD34^{+}) mSCs, activated (CD45^{-}CD31^{-}Sca1^{-}α7i^{+}CD34^{-}) mSCs and FAPs (CD45^{-}CD31^{-}Sca1^{+}α7i^{-}CD34^{+}).

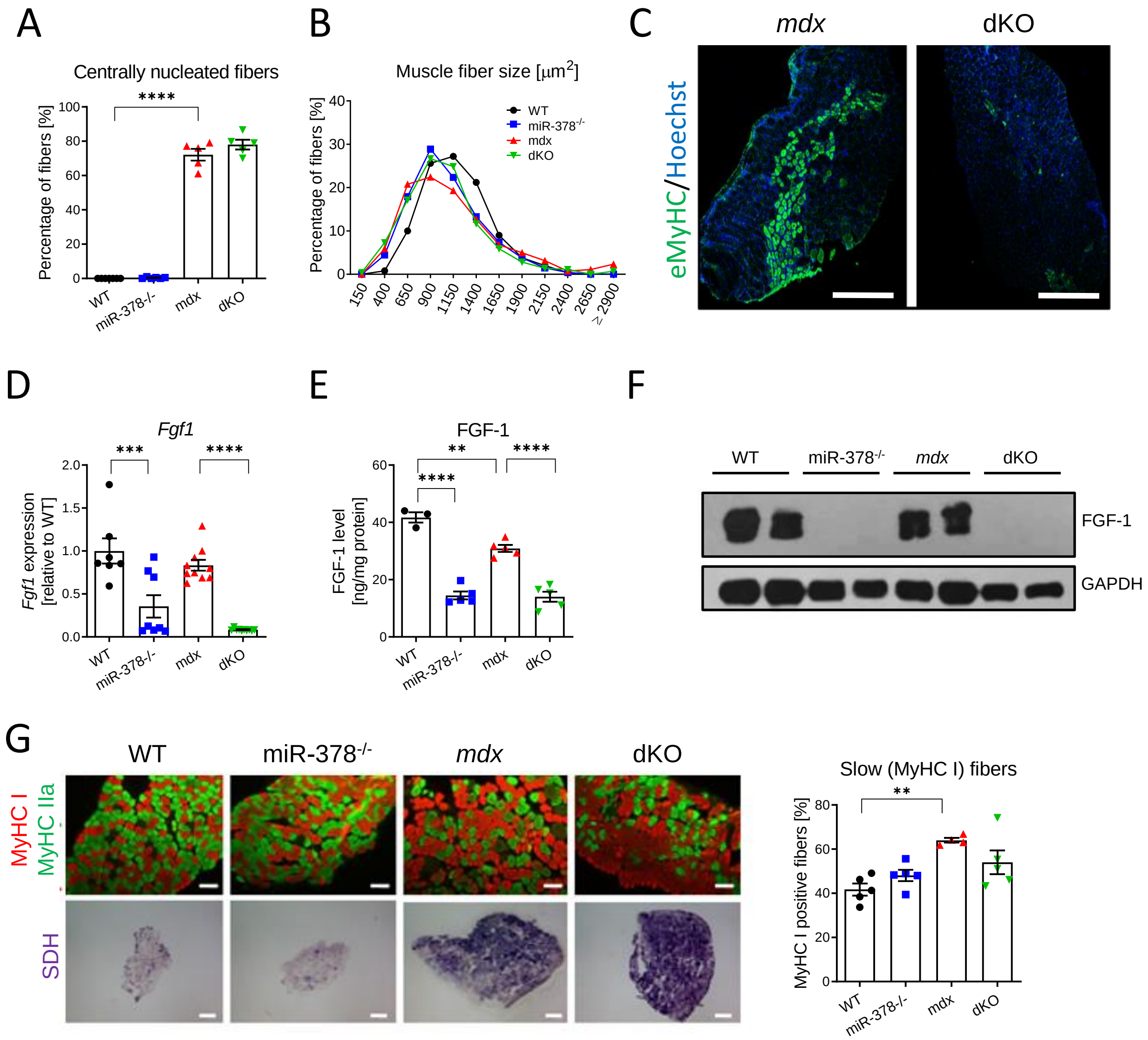

Supplementary Figure 3. The effect of miR-378 deficiency in the soleus muscle of 3-month-old mice. (A) The analysis of centrally nucleated fibers performed based on H&E staining; microscopic assessment using Nikon Eclipse microscope; n=5-7/group. **(B)** The quantification of muscle fibers size based on laminin staining indicating no effect of miR-378 deficiency; n=4-5/group. **(C)** Immunofluorescent staining of eMyHC expression (green) showing its decreased level in dKO mice; Hoechst (blue) was used to visualize nuclei; representative pictures; confocal microscope (LSM-510; Carl Zeiss); scale bar: 100 μm; n=4-5/group. The expression of **(D)** *Fgf1* mRNA and FGF-1 protein level analyzed by **(E)** ELISA and **(F)** Western blot indicating a potent downregulatory effect of miR-378 deficiency; qRT-PCR results calculated as the relative expression level compared to WT animals; n=7-10/group, ELISA test calculated as ng of FGF-1 per mg protein; n=3-5/group; representative Western Blot result. **(G)** Representative pictures of immunofluorescent staining of slow (MyHC I, red), fast (MyHC IIa, green) MyHC isoforms (upper panel) with a histochemical assessment of SDH activity (bottom panel) indicating the increased abundance of oxidative fibers in *mdx* mice; n=4-5/group, microscopic assessment using Nikon Eclipse microscope, scale bar: 100 μm. Data are presented as mean ± SEM. *p<0.05; **p<0.01; ***p<0.001; ****p<0.0001; one-way ANOVA with Tukey's post-hoc test.

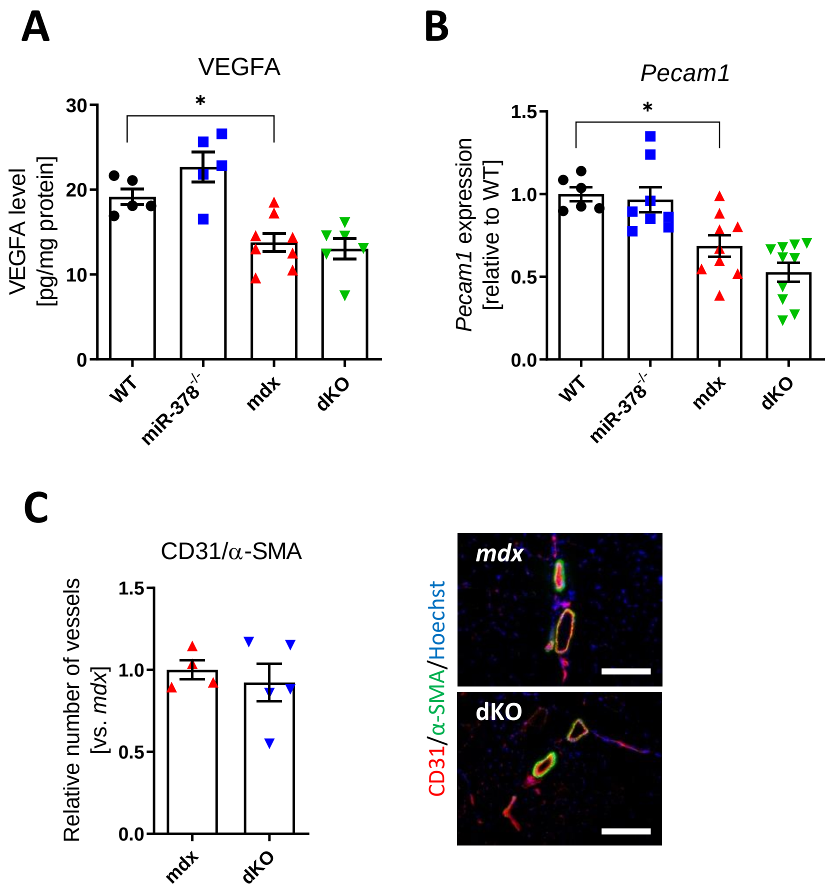

Supplementary Figure 4. miR-378 loss does not affect angiogenesis markers in the muscles of 3-month-old animals. A decreased **(A)** VEGFA protein level in gastrocnemius and **(B)** expression of *Pecam1* in diaphragm muscles of *mdx* animals without any apparent changes driven by the lack of miR-378; ELISA (n=5-8/group) and qRT-PCR (n=6-10/group) analyses, respectively. **(C)** No alterations in the blood vessels abundance in gastrocnemius muscle of dKO vs. *mdx* mice; microscopic assessment using Nikon Eclipse microscope, scale bar: 100 µm. Data are presented as mean ± SEM. *p<0.05; **(A-B)** one-way ANOVA with Tukey's post-hoc test; **(C)** unpaired 2-tailed Student's t-test.

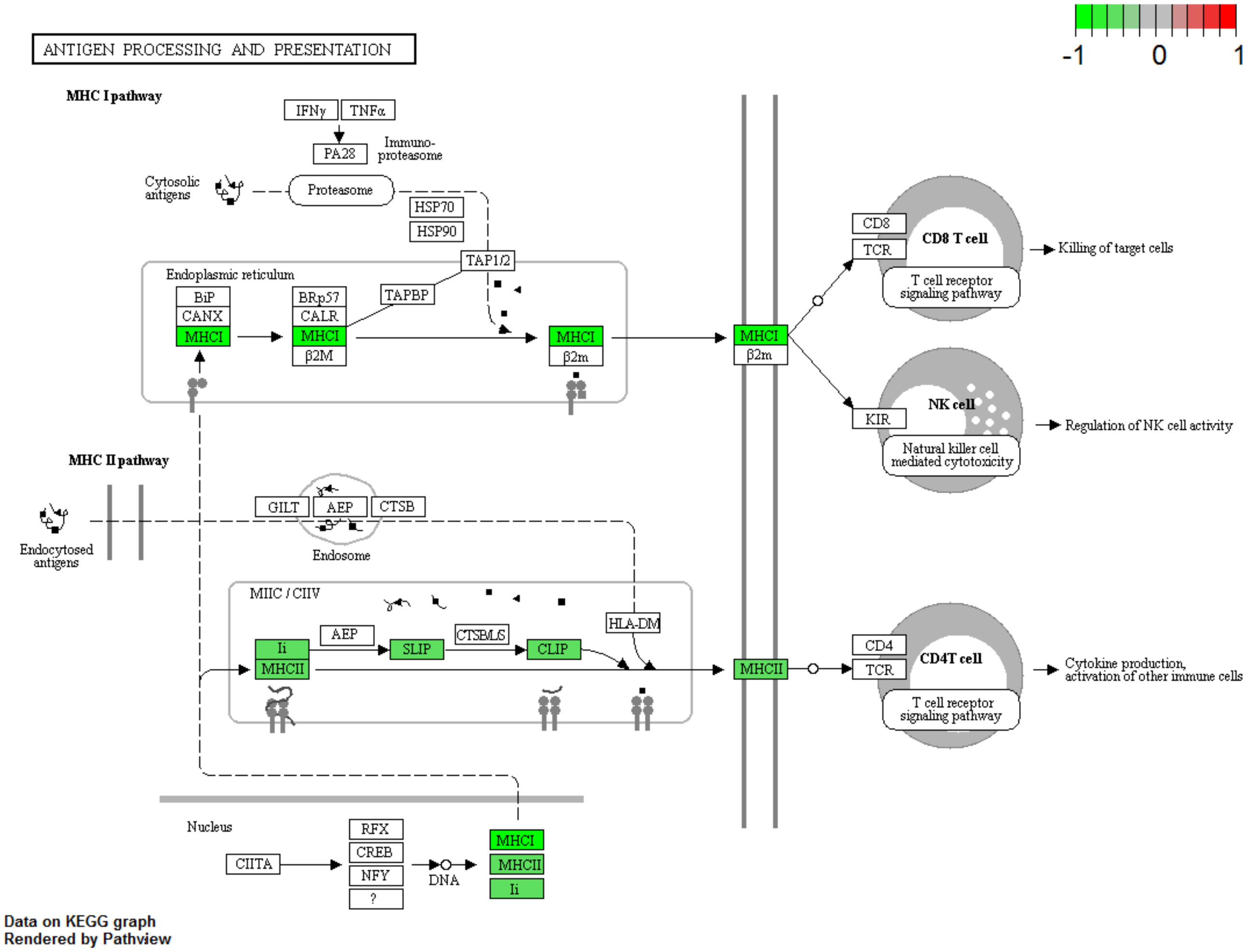

Supplementary Figure 5. KEGG pathway analysis based on RNA-sequencing data. Antigen processing and presentation pathway is diminished in dKO vs. *mdx* mice.
